# Network meta-analysis of the effects of different dietary patterns on patients with metabolic syndrome

**DOI:** 10.3389/fnut.2025.1634545

**Published:** 2025-10-22

**Authors:** Jie Lv, Shiyu Jiao, Wenjing Li, Shuo Ding, Li Ma, Qian Zhang

**Affiliations:** ^1^Shanxi Bethune Hospital, Shanxi Academy of Medical Sciences, Third Hospital of Shanxi Medical University, Tongji Shanxi Hospital, Taiyuan, China; ^2^School of Nursing, Shanxi University of Chinese Medicine, Taiyuan, China

**Keywords:** metabolic syndrome, dietary patterns, randomized controlled trial, dietary interventions, network meta-analysis

## Abstract

**Background:**

Dietary patterns play an important role in the management of metabolic syndrome (MetS). Previous meta-analyses have shown that the ketogenic diet, the Dietary Approaches to Stop Hypertension (DASH) diet, the vegetarian diet, the Mediterranean diet, the low-fat diet, and the low-carbohydrate diet are beneficial for patients with MetS, but there is still a lack of direct comparison of the intervention effects of the above six dietary patterns.

**Objective:**

This study aimed to explore the intervention efficacy of different dietary patterns on MetS and to evaluate and compare the corresponding effects.

**Methods:**

A comprehensive search was carried out in electronic databases such as Embase, Cochrane Library, PubMed, Web of Science, Scopus, CNKI, Wanfang, VIP, and CBM. The search covered studies published from the establishment of the databases up to 1 April 2025, with a focus on dietary patterns that can improve metabolic syndrome. A network meta-analysis was carried out using Stata 16.0 software.

**Results:**

Eventually, 26 randomized controlled trials were included, involving 2,255 patients. The results of the network meta-analysis showed that the DASH diet [MD = −5.72, 95% CI (−9.74, −1.71)] and the vegan diet [MD = −12.00, 95% CI (−18.96, −5.04)] were more effective in reducing the waist circumference of patients with MetS than the control diet group. In terms of lowering blood pressure, the DASH diet [MD = −5.99, 95% CI (−10.32, −1.65)] and the ketogenic diet [MD = −11.00, 95% CI (−17.56, −4.44)] were more effective in reducing systolic blood pressure in patients with MetS than the control diet group. The ketogenic diet [MD = −9.40, 95% CI (−13.98, −4.82)] was more effective in reducing diastolic blood pressure in patients with MetS than the control diet group (*p* < 0.05). According to the ranking results, a vegan diet is the best choice in terms of reducing waist circumference and increasing high-density lipoprotein cholesterol levels. The ketogenic diet is highly effective in lowering blood pressure and triglyceride levels. The Mediterranean diet is highly effective in regulating fasting blood glucose.

**Conclusion:**

The current evidence indicates that the vegan diet, the ketogenic diet, and the Mediterranean diet might have more pronounced effects in ameliorating MetS. Further high-quality research is needed to validate these findings.

**Systematic review registration:**

https://www.crd.york.ac.uk/PROSPERO/, identifier (CRD420251052075).

## Introduction

Metabolic syndrome (MetS) is a chronic non-communicable disorder characterized by a complex array of metabolic abnormalities. These include insulin resistance, hypertension, abdominal obesity, impaired glucose metabolism, and dyslipidemia. In 2009, the International Diabetes Federation (IDF) proposed that MetS can be diagnosed when any three of the following five risk factors are present: abdominal obesity, elevated blood pressure, elevated triglycerides, decreased high-density lipoprotein cholesterol, and elevated fasting glucose are widely used diagnostic criteria (1, 2). In recent years, MetS has become an important global public health issue. Epidemiological data suggest that the global prevalence of MetS in adults has exceeded 20%, and the incidence continues to increase over time (3). From the perspective of regional distribution, the prevalence of metabolic syndrome in Chinese adults reported in relevant literature from 2014 to 2017 was 21.9% (4). The prevalence of MetS in the adult population has increased from 20 to 25% in developed countries, and its incidence continues to increase over time (5–7). Furthermore, MetS not only doubles the risk of type 2 diabetes and quintuples the risk of major cardiovascular events but also significantly elevates the risk of other chronic diseases, including cancer, neurodegenerative diseases, non-alcoholic fatty liver disease, and circulatory system diseases (8–10).

Dietary factors have emerged as a crucial predictor of the incidence and progression of chronic diseases. Traditional methods in nutritional epidemiology primarily analyze the individual impact of food or nutrients on health (11). However, diet is a complex blend of diverse foods, and different foods and nutrients are interconnected and interact with one another. Analyzing the relationship between a single food or nutrient and health fails to fully reflect the comprehensive impact of diet on health. Therefore, in recent years, scholars both at home and abroad have put forward and established a comprehensive evaluation index for the intake status of individual foods and nutrients based on modern nutritional epidemiology methods—dietary patterns. This index not only takes into account the individual effects of nutrients and foods but also considers the interactions between them, enabling a more comprehensive assessment of the impact of diet on health. Previous meta-analyses have verified that the ketogenic diet pattern exerts a significant influence on metabolic factors such as body mass index (BMI), high-density lipoprotein cholesterol (HDL-C), triglyceride (TG), and blood glucose in patients with MetS (12). The Dietary Approaches to Stop Hypertension (DASH) diet has been validated for its beneficial effects on blood pressure and lipid profiles, significantly reducing both systolic and diastolic blood pressure levels while modulating concentrations of high-density lipoprotein cholesterol (HDL-C), low-density lipoprotein cholesterol (LDL-C), and total cholesterol (13). The vegan diet pattern has been shown to reduce weight and blood glucose levels in patients with MetS (14). The Mediterranean diet pattern exerts a beneficial impact on the incidence of MetS (15). The low-fat diet pattern has the potential to reduce C-reactive protein in patients with MetS (16). The low-carbohydrate diet pattern has the potential to reduce weight and blood glucose levels in patients with MetS. Although dietary patterns have gradually become the primary focus of dietary guidelines, the comparison of dietary patterns for primary prevention in MetS remains limited. At present, in the academic community, there is a dearth of direct comparisons of the intervention effects of the six dietary patterns mentioned above. Network meta-analysis is capable of integrating direct and indirect evidence and ranking the effects of diverse intervention methods according to the analysis findings. Therefore, this study aims to utilize network meta-analysis to synthesize the actual effects of different dietary patterns in treating patients with MetS and compare their efficacies. This will provide the most suitable dietary pattern for MetS patients and comprehensive evidence for the future clinical treatment of MetS patients.

## Methods

The present systematic review and NMA were performed according to the Preferred Reporting Items for Systematic Reviews and Meta-analysis Network Meta-analysis Extension Statement (17). This research has been registered on the International Prospective Register of Systematic Reviews (PROSPERO). Registration number: CRD420251052075.

### Inclusion criteria

1. Population (P): Patients who have been diagnosed with MetS and are 18 years of age or older. There are no restrictions regarding nationality, race, gender, or disease duration.
2. Intervention (I): The observation group selected one of six dietary patterns, namely the DASH diet, vegan diet, low-carbohydrate diet, Mediterranean diet, low-fat diet, and ketogenic diet.
  (1) DASH diet: A dietary pattern with high intake of fruits, vegetables, low-fat dairy products, and whole grains and limited intake of red meat and sugar. Fat accounts for 27% (saturated fat 6%), carbohydrate 55%, and protein 18% (18).
  (2) Vegan diet: A dietary pattern with whole grains, legumes, vegetables, fruits, nuts, mushrooms, and algae as the core, with appropriate eggs and milk. The main fat is unsaturated fatty acid, and the ratio of carbohydrate to protein is flexible (19).
  (3) Low-carbohydrate diet: A dietary pattern that strictly limits carbohydrate intake to less than 25% of total energy intake (18).
  (4) Mediterranean diet: A dietary pattern that includes vegetables, fruits, nuts, legumes, whole grains, and olive oil, with moderate amounts of fish, dairy products, and red wine, and limited red meat and processed foods. Fat accounts for 35–45%, mainly from monounsaturated fat, carbohydrate 40–45%, and protein 15–18% (18).
  (5) Low-fat diet: A dietary pattern that emphasizes the intake of high amounts of grains and cereals. Fat accounts for less than 30% of total energy intake, carbohydrate 50–60%, and protein 10–15% (18).
  (6) Ketogenic diet: A dietary pattern that limits carbohydrate intake to 5–10% of total energy intake, and the remaining carbohydrate is replaced by dietary fat and adequate protein (20).
3. Comparison (C): The control diet was used as our reference diet, including the “usual diet” (e.g., the usual diet with no changes) or the “typical national diet”.
4. Outcomes (O): Outcome indicators include waist circumference (WC), systolic blood pressure (SBP), diastolic blood pressure (DBP), fasting blood glucose (FBG), triglycerides (TG), and high-density lipoprotein cholesterol (HDL-C).
5. Study design (S): The study design was a randomized controlled trial (RCT).

### Exclusion criteria

The exclusion criteria were as follows:

1. Children (aged <18 years), pregnant women, or lactating women,
2. Duplicate publications,
3. Literature not in Chinese or English, and
4. Literature for which the original text or raw data could not be retrieved.

### Search strategy

Computerized searches were performed in databases including EMBASE, Cochrane Library, PubMed, Web of Science, Scopus, China National Knowledge Infrastructure (CNKI), Wanfang Data Knowledge Service Platform, VIP Chinese Science and Technology Journal Database (VIP), and China Biomedical Literature Database (CBM). The search was carried out by integrating MeSH subject terms and free terms. The search strategy was modified in accordance with the search rules of different databases. The snowball approach was used to manually search for relevant references containing the literature and to keep track of the relevant studies that have been incorporated into the systematic review. The search period spanned from the establishment of the databases to 1 April 2025. The search strategy for PubMed is presented in Table 1.

**Table 1:** Search strategy in PubMed.

Steps	Search
#1	(metabolic syndrome[Title/Abstract])OR (Metabolic Syndrome[MeSH Terms])
#2	(((((((((((((((((((((((((((diet*[Title/Abstract]) OR (dietary pattern*[Title/Abstract])) OR (Diet, Ketogenic[MeSH Terms])) OR (Ketogenic diet*[Title/Abstract])) OR (Keto diet*[Title/Abstract])) OR (Diet, DASH[MeSH Terms])) OR (DASH diet*[Title/Abstract])) OR (Dietary Approaches to Stop Hypertension[Title/Abstract])) OR (DASH eating plan[Title/Abstract])) OR (Diet, Vegetarian[MeSH Terms])) OR (Vegetarian diet*[Title/Abstract])) OR (Vegan diet*[Title/Abstract])) OR (plant-based diet*[Title/Abstract])) OR (meat-free diet*[Title/Abstract])) OR (Diet, Mediterranean[MeSH Terms])) OR (Mediterranean diet*[Title/Abstract])) OR (Mediterranean dietary pattern[Title/Abstract])) OR (MedDiet[Title/Abstract])) OR (cretan diet[Title/Abstract])) OR (Diet, Fat-Restricted[MeSH Terms])) OR (Low-fat diet*[Title/Abstract])) OR (fat-restricted diet*[Title/Abstract])) OR (reduced-fat diet*[Title/Abstract])) OR (hypolipidemic diet*[Title/Abstract])) OR (Diet, Carbohydrate-Restricted[MeSH Terms])) OR (Low-carbohydrate diet*[Title/Abstract])) OR (low-carb diet*[Title/Abstract])) OR (carb-restricted diet*[Title/Abstract])
#3	((((Randomized Controlled Trials[Publication Type]) OR (controlled clinical trial[Publication Type])) OR (randomized[Title/Abstract])) OR (randomly[Title/Abstract]))
#4	#1 AND #2 AND #3

### Literature screening and data

Two trained researchers (JL and SJ) independently performed the initial screening and full-text screening of the literature. Any discrepancies were resolved through discussion or with the involvement of a third researcher (QZ) when necessary. Data extraction was conducted separately by JL and subsequently cross-verified by QZ to ensure accuracy. The literature was imported into EndNote X9, a literature management software. Duplicate and irrelevant literature were removed, and the abstracts and full texts were then read. The literature was screened in accordance with the inclusion and exclusion criteria, and relevant information was retrieved. The extracted information included details of the underlying study, which included the name of the first author, year of publication, country, sample size in each group, intervention method, proportion of women, age, duration of the intervention, outcome measures, and energy restriction and drop-out rates.

### Literature quality assessment

Two researchers used the Cochrane Risk of Bias in Randomized Trials (RoB 2) tool (21) to assess the included literature. The evaluation content covered: randomization process, bias in intervention implementation, missing outcome data, outcome measurement, and selection of reported results. According to the RoB 2 results, each article was categorized as “high risk,” “some concerns,” or “low risk.” Disagreements were addressed through consultation with other members of the research team.

### Statistical approaches

This research used network meta-analysis to assess the disparities in the impacts of various dietary patterns on outcome indicators. The analysis was carried out using Stata 16.0 software. The direct comparative relationship and closed loop among the intervention measures were visualized by drawing a mesh diagram. The size of the node and the width of the edge represent the sample size, weight, and number of direct comparative studies, respectively. The consistency of evidence is assessed through the global chi-squared test. The mean difference (MD) was used as an effect index for continuous variables. A league table was constructed using the random effects model. The MD values and their 95% confidence intervals (CIs) for all pairwise comparisons were presented in matrix format. A *p*-value of less than 0.05 indicated that the difference was statistically significant. When a study had two dietary pattern interventions, they were treated as separate studies and were included in the NMA. The area under the surface of the cumulative ranking curve (SUCRA) of various intervention methods was quantified and presented graphically. The probability of each dietary pattern emerging as the best choice was evaluated and characterized. Finally, the funnel plot was utilized to assess publication bias.

### Sensitivity analyses

A sensitivity analysis was performed to assess the potential effect of the duration of the intervention on the robustness of the pooled results. The analysis restricted the included studies to those with intervention durations between 12 and 48 weeks to rule out possible heterogeneity introduced by very short or long intervention periods and to test the reliability of the main conclusions. Within this range, we reconstructed the evidence network for all prespecified outcome measures and used a random-effects model that was consistent with the main analysis to perform a network meta-analysis to determine the sensitivity of the results to intervention duration by comparing the effect size estimates with the cumulative SUCRA values.

## Results

### Literature search results

**Figure 1:**
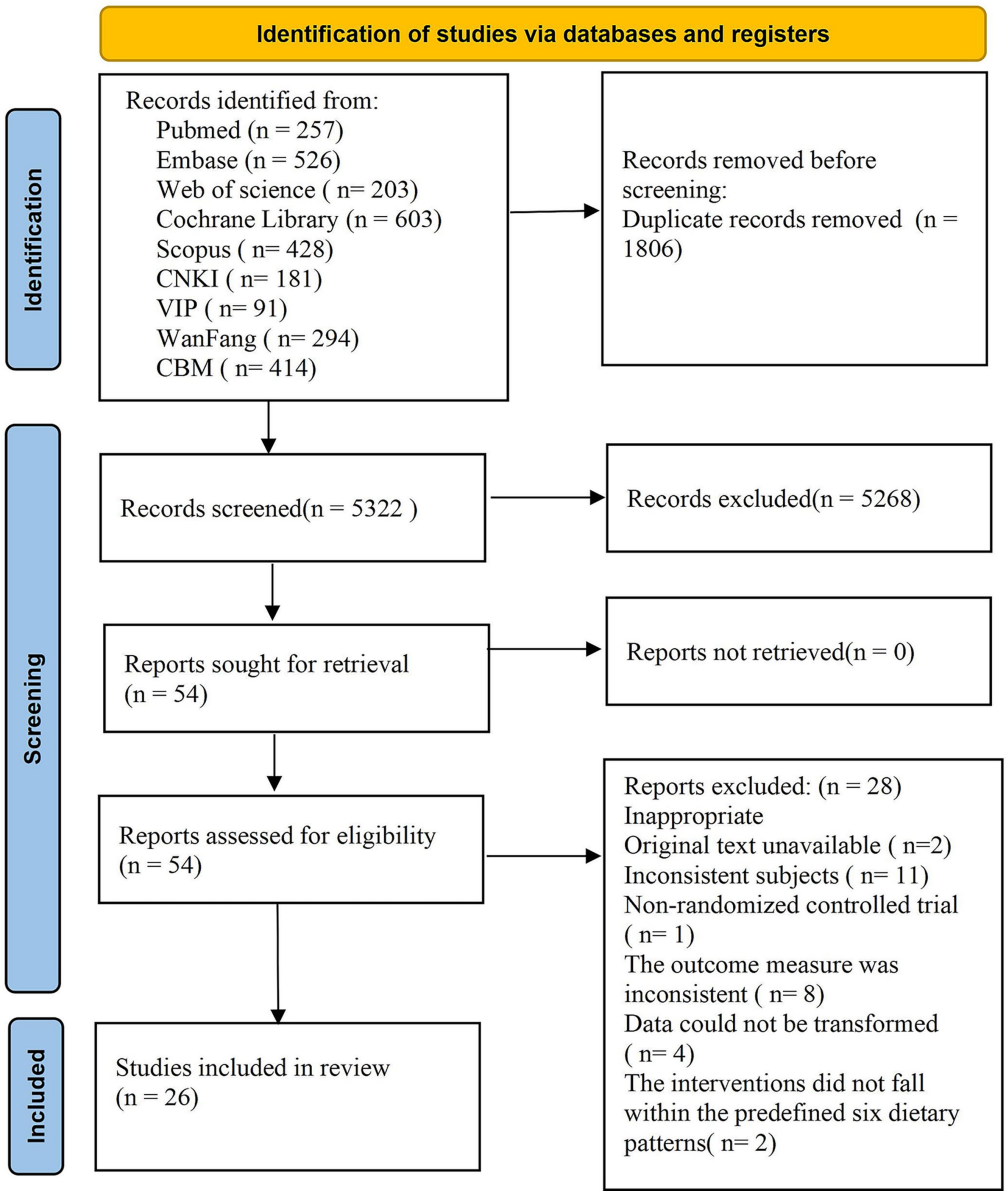
Flow diagram of included studies.

A total of 7,128 articles were retrieved from the database, with 1,806 duplicate articles excluded. Subsequently, 5,268 articles were eliminated based on a review of their titles and abstracts, and finally, 54 articles met the inclusion criteria. Upon further examination of the full texts, an additional 28 articles were excluded: specifically, 2 articles could not be obtained, 11 were excluded due to inconsistent research subjects, 1 was not an RCT, 8 outcome indicators did not satisfy the inclusion criteria, data from 4 studies could not be converted, and 2 interventions did not align with the predefined 6 dietary patterns. In conclusion, a total of 26 articles were included in the quantitative analysis. The literature screening process and results are illustrated in Figure 1.

### Fundamental characteristics of the included literature

A total of 26 studies (22–47) were incorporated into this research. These studies involved 2,255 patients from 15 countries, namely the United States, Korea, Iran, Poland, Brazil, New Zealand, the United Kingdom, Thailand, Norway, Italy, Israel, Canada, Australia, Ireland, and Spain. The duration of the studies spanned from 4 to 96 weeks, with 14 of them lasting from 12 to 24 weeks. Seven dietary patterns were included: DASH diet, vegan diet, ketogenic diet, low-carbohydrate diet, Mediterranean diet, low-fat diet, and control diet. The basic characteristics of the included literature are shown in Table 2.

**Table 2:** Basic characteristics of the included literature.

Included study	Country	Year	Sample size (example)		% of female		Age		Intervention time (months)	Intervention method		Outcome indicator	Energy restriction	% of drop-out	
			Experimental group	Control group	Experimental group	Control group	Experimental group	Control group		Experimental group	Control group			Experimental group	Control group
Choi et al. (22)	Korea	2015	21	18	100	100	73.0 ± 3.9	73.8 ± 5.8	8 weeks	DASH diet	Control diet (regular diet without special guidance)	a, b, c, d, e	No	0	14.3
Al-Solaiman et al. (23)	The United States	2010	15	15	80	80	40.3 ± 1.7	36.7 ± 1.8	6 weeks	DASH diet	Control diet (regular diet without special guidance)	c, d, e	Yes (the two groups were calorically equal)	21	17
Azadbakht et al. (24)	Iran	2005	28	27	70	70	41.5 ± 12.5	41.3 ± 12.18	24 weeks	DASH diet	Control diet (regular diet without special guidance)	a, b, d	Yes (a daily energy deficit of 500 kcal)	0	0
Kucharska et al. (25)	Poland	2018	64	62	53	48	61.34 ± 7.90	58.11 ± 8.52	12 weeks	DASH diet	Control diet (regular diet without special guidance)	a, b, e	No	The overall dropout rate was 7.2%	
Paula et al. (26)	Brazil	2015	20	20	40	70	61.8 ± 8.1	62.5 ± 8.8	4 weeks	DASH diet	Control diet (regular diet without special guidance)	a, b, d, e	Yes (25–30 kcal/kg)	0	0
Wright et al. (27)	New Zealand	2017	33	32	67	53	56 ± 9.9	56 ± 9.5	24 weeks	Vegan diet	Control diet (regular diet without special guidance)	a,b,d	No	24	25
Mishra et al. (28)	The United States	2013	142	149	77	88	44.3 ± 15.3	44.3 ± 15.3	18 weeks	Vegan diet	Control diet (regular diet without special guidance)	b, c, d	No	34	21
Barnard et al. (29)	The United States	2020	30	32	73	81	56.6 ± 10.9	58.3 ± 8.4	16 weeks	Vegan diet	Mediterranean diet	c, d, e	No	The overall dropout rate was 16%	
Kahleova et al. (30)	The United States	2020	117	106	/		53 ± 10	57 ± 13	16 weeks	Vegan diet	Control diet (regular diet without special guidance)	c,d,e	/	4.1	13.1
Volek et al. (31)	The United States	2009	20	20	50	50	32.6 ± 11.3	36.9 ± 12.5	12 weeks	Ketogenic diet	Low-fat diet	c, d, e	Yes (1,500 kcal/day)	0	0
Bradley et al. (32)	the United Kingdom	2009	12	12	58	67	37.1 ± 8.9	40.5 ± 10.4	8 weeks	Low-carbohydrate diet	Low-fat diet	a, b, c, d, e	Yes (a daily energy deficit of 500 kcal)	11	11
Volek et al. (33)	The United States	2004	13	13	100	100	34.0 ± 8.6		4 weeks	Low-carbohydrate diet	Low-fat diet	c,d,e	/	0	0
Pinsawas et al. (34)	Thailand	2017	26	22	73	86	40.9 ± 1.7	38.5 ± 1.7	52 weeks	Ketogenic diet	Control diet (typical Thai diet)	a, b	/	/	
Saslow et al. (35)	The United States	2017	16	18	/		/		48 weeks	Low-carbohydrate diet	Low-fat diet	b,c,d	Yes (the experimental group had a daily reduction of 500 kcal and the control group had no change)	b, c, d	17
Veum et al. (36)	Norway	2017	20	16	0	0	40.3 ± 5.53	40.2 ± 4.50	12 weeks	Low-carbohydrate diet	Low-fat diet	b, d, e	/	11	20
Esposito et al. (37)	Italy	2004	90	90	50	40	44		96 weeks	Mediterranean diet	Control diet (regular diet without special guidance)	a,b,c,d,e	No	8.9	8.9
Elhayany et al. (38)	Israel	2010	63	55	44	50	55–57		48 weeks	Mediterranean diet	Control diet (regular diet without special guidance)	a,c,d,e	Yes (The two groups were calorically equal)	The overall dropout rate was 25.1%	
Richard et al. (39)	Canada	2011	26	26	0	0	49		20 weeks	Mediterranean diet	Control diet (regular diet without special guidance)	a,b,c,d,e	Yes (Reduce 500 kcal daily)	The overall dropout rate was 10.3%	
Fortin et al. (40)	Canada	2018	14	14	50	36	52.1 ± 9.7	49.8 ± 11.2	24 weeks	Mediterranean diet	Low-fat diet	a, b, c, d, e	No	10.3	0
Wade et al. (41)	Australia	2018	19	19	68		60.8 ± 6.3	59.6 ± 7.6	8 weeks	Mediterranean diet	Low-fat diet	b,d,e	Yes (About 2000 kcal/ day)	9	9.1
Sofi et al. (42)	Italy	2018	103	104	78		50		12 weeks	Mediterranean diet	Vegan diet	c,d,e	No	12	13
Tierney et al. (43)	Ireland	2010	106	100	54	53	54.70 ± 0.91	54.91 ± 0.86	12 weeks	Low-fat diet	Control diet (typical Nordic diet)	a, b, e	No	10.9	17.4
Meneses et al. (44)	Spain	2011	12	8	/		56.5 ± 2.0	57.8 ± 3.1	12 weeks	Low-fat diet	Control diet (typical Nordic diet)	c,d,e	Yes (About 2380 kcal/ day)	/	
Cruz-Teno et al. (45)	Spain	2012	20	17	65	64	56.3 ± 1.8	58.5 ± 1.9	12 weeks	Low-fat diet	Control diet	b, c, d	Yes (approximately 1,960 kcal/day)	0	0
Camhi et al. (46)	The United States	2010	26	25	/		40.2 ± 4.5	40.3 ± 5.5	8 weeks	Low-fat diet	Low-carbohydrate diet	a,b,d	Yes (About 2205 kcal/day)	0	0
Fernandez et al. (47)	The United States	2021	96	96	80	80	51.9 ± 13.1	52.8 ± 13.2	12 weeks	Low-fat diet	Low-carbohydrate diet	a, b, c, d, e	Yes (the experimental group had a daily reduction of 500 kcal and the control group had no change)	19.8	16.7

### Bias risk in the included studies

**Figure 2:**
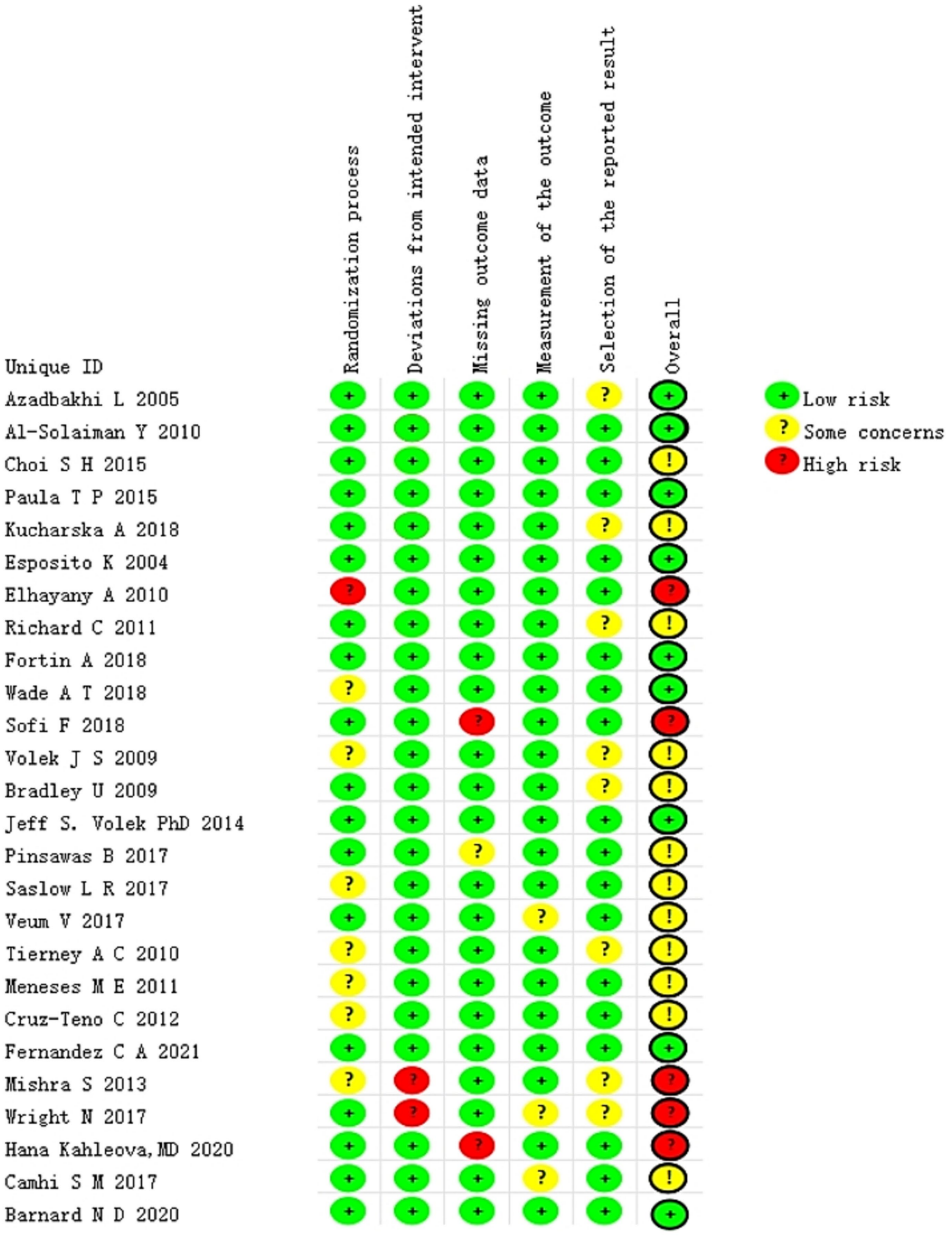
Summary of risk bias.

All of the included studies were evaluated independently and simultaneously by two authors. The findings of the risk of bias (RoB) analysis are presented in Figure 2. The overall risk of bias (RoB) of five studies (27, 28, 30, 38, 42) was rated as high, whereas that of 21 studies (22–26, 29, 31–37, 39–41, 43–47) was rated as low. Among the five types of risks assessed, namely randomization process, deviations from intended intervention(s), missing outcome data, outcome measurement, and outcome reporting selection, the first two were the primary sources of bias in the included studies.

In the evaluation of the randomization process, if the study report was lacking key details or explicitly stated that double blinding had failed, it was determined that a bias risk existed in this area. The crucial aspects of assessing deviations from the intended intervention(s) include the blind execution by assessors or analysts, as well as potential systematic errors that could be introduced by the experimental environment. Regarding the issue of missing outcome data, a majority of studies reported the data of randomized subjects either completely or almost completely. We have considered a dropout rate of ≤10% as one of our criteria. Conversely, studies identified as having problems or a high risk of bias often had a substantial proportion of subjects drop out or did not report any information regarding missing data whatsoever. The quality assessment findings of the included literature are presented in Figure 2. In most studies, the risk of outcome measurement bias was judged to be low, as the assessments were conducted by an independent third party not involved in the research. However, two studies constituted exceptions. One study (36) provided insufficient information regarding whether outcome assessors were aware of the participants’ assigned interventions, thereby introducing uncertainty about potential bias. In the other study (27), the researchers responsible for measuring outcomes were explicitly aware of the participants’ group allocation, which may have compromised the objectivity of the results due to prior knowledge of treatment assignment. Furthermore, eight studies were assessed as having a moderate risk of selective outcome reporting due to the absence of a pre-specified trial protocol. Consequently, we categorized this domain as “some concerns.” It should also be noted that this study only addressed implementation bias in relation to “blinding” for those studies deemed at risk. The primary reason for this limitation lies in the inherent characteristics of dietary interventions—specific intervention strategies, such as modifications in food intake or dietary structure, are difficult to conceal from participants and caregivers, thereby making blinding particularly challenging.

### Network evidence diagram

**Figure 3:**
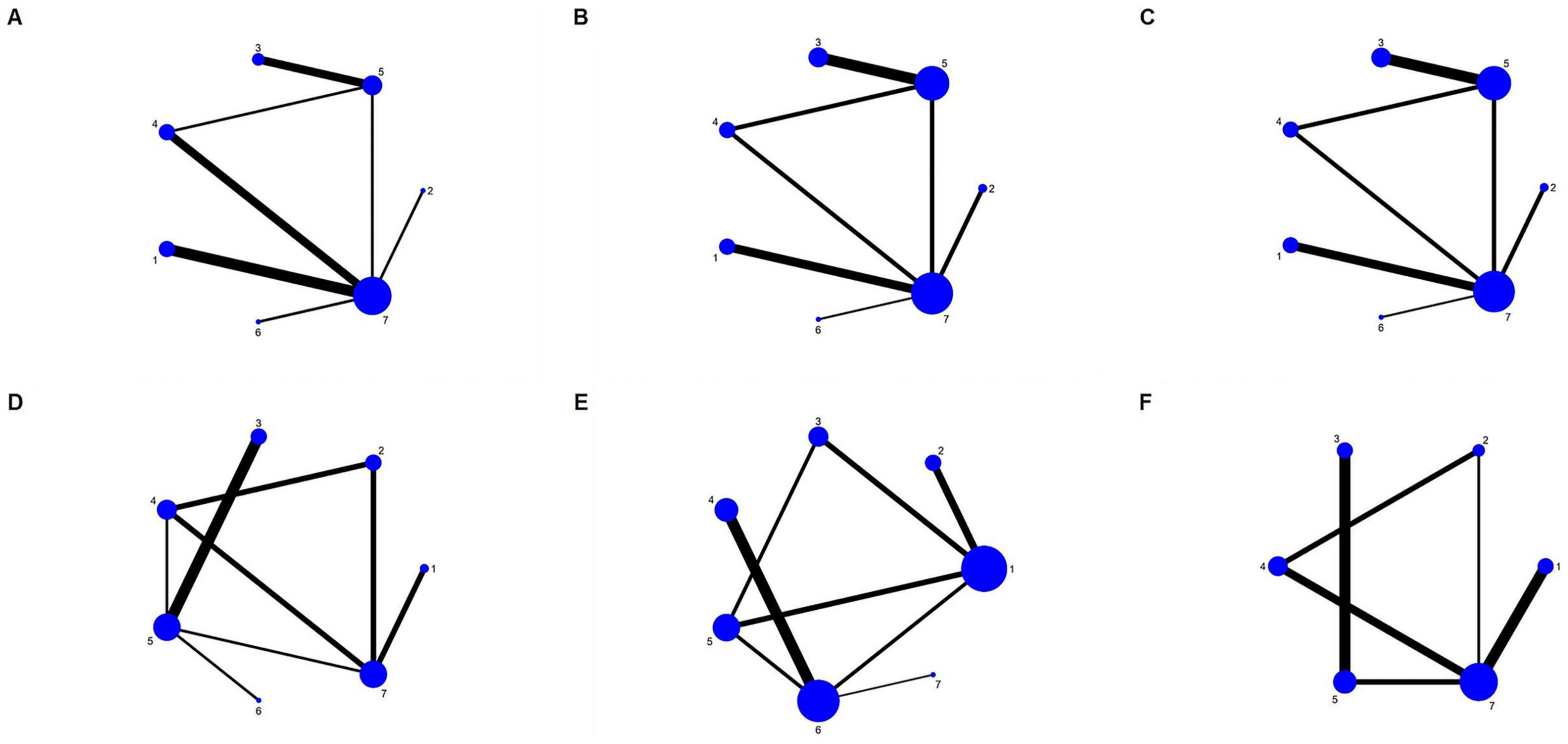
Network diagram for each outcome. **(A)** WC; **(B)** SBP; **(C)** DBP; **(D)** TG; **(E)** HDL-C; **(F)** FBG. 1, DASH diet; 2, vegan diet; 3, low-carbohydrate diet; 4, Mediterranean diet; 5, low-fat diet; 6, ketogenic diet; and 7, control diet.

Figure 3 presents a network diagram of direct comparisons for each outcome indicator. Each dot in the figure corresponds to a distinct dietary pattern. The size of the dots reflects the sample size incorporated in each dietary pattern. The lines that connect the dots signify direct comparisons among dietary patterns. The thickness of these lines is proportional to the number of RCTs included in each pair of dietary patterns. A total of 14 articles (22, 24–27, 32, 34, 37–40, 43, 46, 47) presented findings on WC, covering all seven dietary patterns. Eighteen articles (22, 24–28, 32, 34–37, 39–41, 43, 45–47) reported on blood pressure, covering all seven dietary patterns. Seventeen articles (22, 23, 28–33, 35, 37–42, 44, 45, 47) reported findings regarding TG, encompassing all seven dietary patterns. Twenty-three articles (22–24, 26–33, 35–42, 44–47) presented findings on high-density lipoprotein cholesterol, covering all seven dietary patterns. Nineteen articles (22, 23, 25, 26, 29–33, 36–44, 47) reported on the impact of FBG, involving six dietary patterns.

### Consistency check

The findings of the consistency check for this study indicate that the *p*-value for each model is greater than 0.05. Specifically, the p-value for the WC score is 0.276, for the SBP score is 0.499, for the DBP score is 0.536, for the triglyceride score is 0.624, for the high-density lipoprotein cholesterol score is 0.089, and for the FBG score is 0.463. Consequently, the overall consistency is satisfactory.

### Results of network meta-analysis on WC

The findings of the network meta-analysis revealed that, in comparison to the control diet group, the DASH diet [MD = −5.72, 95% CI (−9.74, −1.71)] and the vegan diet [MD = −12.00, 95% CI (−18.96, −5.04)] significantly decreased the WC of patients with MetS. Additionally, compared to the low-carbohydrate diet [MD = −10.34, 95% CI (−20.60, −0.07)], the Mediterranean diet [MD = −10.67, 95% CI (−18.80, −2.54)], the low-fat diet [MD = −10.77, 95% CI (−19.76, −1.79)], and the ketogenic diet [MD = −9.79, 95% CI (−19.44, −0.14)], the vegan diet led to a greater reduction in WC. The detailed results are presented in Table 3.

**Table 3:** League table of all pairwise comparisons of the effects of dietary patterns on WC in MetS patients.

WC						
**DASH diet**						
6.28 (−1.76, 14.31)	**Vegan diet**					
−4.06 (−12.57, 4.45)	**−10.34 (−20.60, −0.07)**	**Low-carbohydrate diet**				
−4.39 (−10.21, 1.43)	**−10.67 (−18.80, −2.54)**	−0.33 (−8.30, 7.63)	**Mediterranean diet**			
−4.50 (−11.39, 2.39)	**−10.77 (−19.76, −1.79)**	−0.44 (−5.49, 4.61)	−0.10 (−6.34, 6.14)	**Low-fat diet**		
−3.52 (−11.31, 4.28)	**−9.79 (−19.44, −0.14)**	0.54 (−9.53, 10.62)	0.88 (−7.01, 8.77)	0.98 (−7.78, 9.75)	**Ketogenic diet**	
**−5.72 (−9.74, −1.71)**	**−12.00 (−18.96, −5.04)**	−1.66 (−9.21, 5.88)	−1.33 (−5.52, 2.86)	−1.23 (−6.90, 4.45)	−2.21 (−8.89, 4.48)	**Control diet**

Statistically significant treatment effects are in bold.

**Figure 4:**
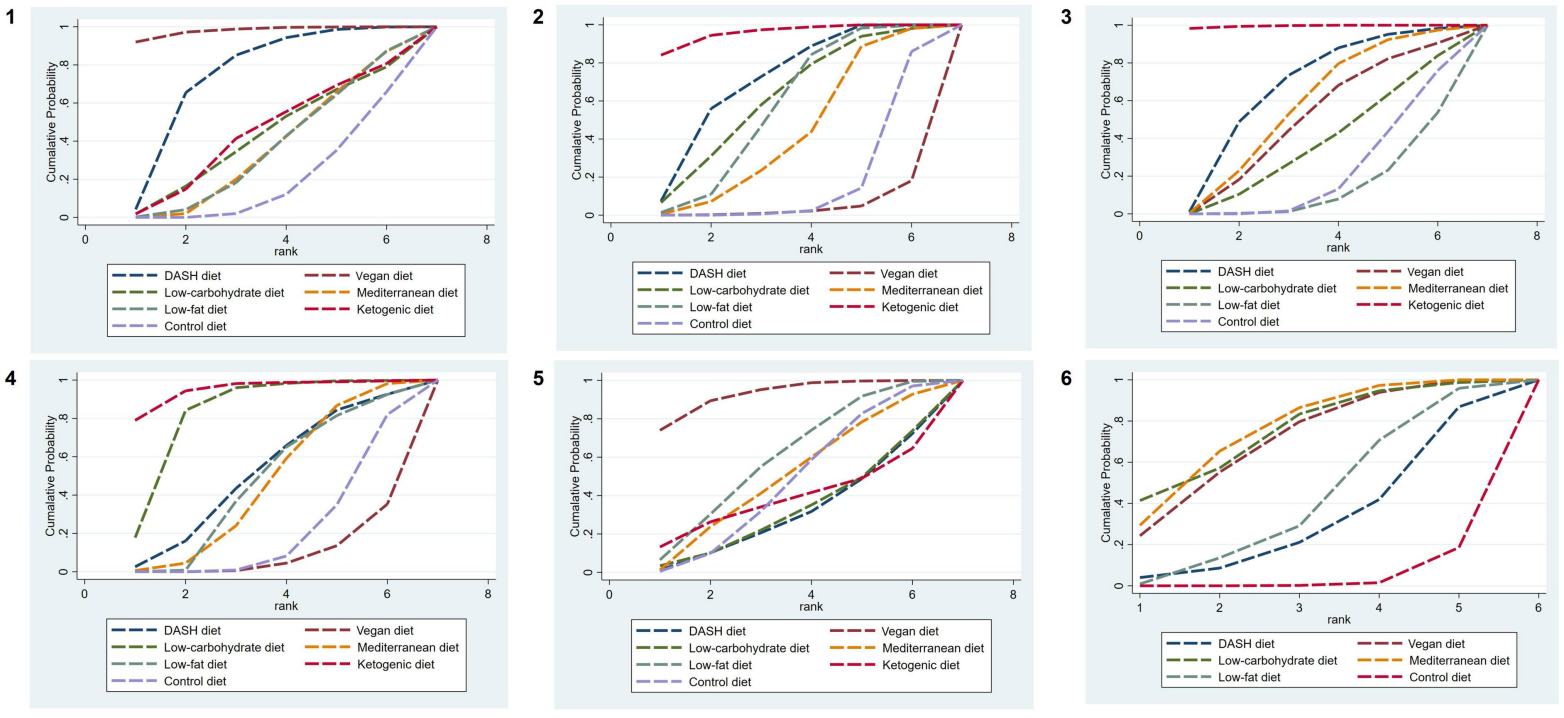
SUCRA plots of different outcome indicators in MetS patients treated with different dietary patterns. **1**, WC; **2**, SBP; **3**, DBP; **4**, TG; **5**, HDL-C; and **6**, FBG.

The SUCRA ranking results show that the vegan diet (SUCRA = 97.9%) > DASH diet (SUCRA = 74.6%) > ketogenic diet (SUCRA = 43.9%) > low-carbohydrate diet (SUCRA = 42.0%) > Mediterranean diet (SUCRA = 36.2%) = low-fat diet (SUCRA = 36.2%) > control diet (SUCRA = 19.2%). The ranking outcomes of each dietary pattern are presented in Figure 4.

### Results of network meta-analysis on SBP

The findings of the network meta-analysis revealed that, in comparison with the control diet group, the DASH diet [MD = −5.99, 95% CI (−10.32, −1.65)] and the ketogenic diet [MD = −11.00, 95% CI (−17.56, −4.44)] significantly decreased the SBP of patients with MetS. Additionally, compared with the vegan diet [MD = 13.38, 95% CI (5.25, 21.52)] and the Mediterranean diet [MD = 8.13, 95% CI (0.22, 16.04)], the ketogenic diet led to a more significant reduction in SBP. The detailed findings are presented in Table 4.

**Table 4:** League table of all pairwise comparisons of the effects of dietary patterns on SBP in MetS patients.

SBP						
**DASH diet**						
−8.37 (−14.86, −1.88)	**Vegan diet**					
−1.45 (−8.64, 5.75)	6.92 (−0.69, 14.54)	**Low-carbohydrate diet**				
−3.12 (−9.36, 3.12)	5.25 (−1.27, 11.76)	−1.67 (−7.97, 4.62)	**Mediterranean diet**			
−1.88 (−7.83, 4.07)	6.49 (−0.06, 12.91)	−0.43 (−4.46, 3.59)	1.24 (−3.54, 6.02)	**Low-fat diet**		
5.01 (−2.85, 12.87)	**13.38 (5.25, 21.52)**	6.46 (−2.33, 15.25)	**8.13 (0.22, 16.04)**	6.89 (−0.90, 14.68)	**Ketogenic diet**	
**−5.99 (−10.32, −1.65)**	2.38 (−2.43, 7.19)	−4.54 (−10.40, 1.31)	−2.87 (−7.29, 1.55)	−4.11 (−8.31, 0.10)	**−11.00 (−17.56, −4.44)**	**Control diet**

Statistically significant treatment effects are in bold.

The results of SUCRA ranking were as follows: the ketogenic diet (SUCRA = 95.8%) > DASH diet (SUCRA = 70.8%) > low-carbohydrate diet (SUCRA = 61.2%) > low-fat diet (SUCRA = 56.9%) > Mediterranean diet (SUCRA = 43.6%) > control diet (SUCRA = 17.2%) > vegan diet (SUCRA = 4.4%).

### Results of network meta-analysis on DBP

The findings of the network meta-analysis revealed that, in comparison to the DASH diet [MD = −6.57, 95% CI (−1.05, −12.09)], vegan diet [MD = −7.80, 95% CI (−2.17, −13.44)], low-carbohydrate diet [MD = −8.87, 95% CI (−2.78, −14.95)], Mediterranean diet [MD = −7.50, 95% CI (−1.96, −13.04)], low-fat diet [MD = −10.01, 95% CI (−4.61, −15.42)], and control diet [MD = −9.40, 95% CI (−13.98, −4.82)], the ketogenic diet demonstrated a more significant reduction in DBP, as presented in Table 5.

**Table 5:** League table of all pairwise comparisons of the effects of dietary patterns on DBP in MetS patients.

DBP						
**Ketogenic diet**						
**−7.80 (−2.17, −13.44)**	**Vegan diet**					
**−8.87 (−2.78, −14.95)**	−1.06 (−6.26, 4.13)	**Low-carbohydrate diet**				
**−7.50 (−1.96, −13.04)**	0.30 (−4.23, 4.83)	1.37 (−2.86, 5.59)	**Mediterranean diet**			
**−10.01 (−4.61, −15.42)**	−2.21 (−6.57, 2.15)	−1.15 (−3.93, 1.63)	−2.51 (−5.69, 0.67)	**Low-fat diet**		
**−6.57 (−1.05, −12.09)**	1.23 (−5.73, 3.27)	2.30 (−7.37, 2.77)	0.93 (−5.32, 3.46)	3.44 (−7.66, 0.77)	**DASH diet**	
**−9.40 (−13.98, −4.82)**	−1.59 (−4.87, 1.69)	−0.53 (−4.53, 3.47)	−1.89 (−5.01, 1.23)	0.62 (−2.25, 3.48)	−2.83 (−5.92, 0.26)	**Control diet**

Statistically significant treatment effects are in bold.

The results of SUCRA ranking were as follows: the ketogenic diet (SUCRA = 99.6%) > DASH diet (SUCRA = 67.5%) > Mediterranean diet (SUCRA = 57.6%) > vegan diet (SUCRA = 50.6%) > low-carbohydrate diet (SUCRA = 37.8%) > control diet (SUCRA = 22.4%) > low-fat diet (SUCRA = 4.4%).

### Results of network meta-analysis on TG

The findings of the network meta-analysis indicated that, when compared with the control diet group, the low-carbohydrate diet [MD = −37.24, 95% CI (−71.13, −3.35)] and the ketogenic diet [MD = −58.66, 95% CI (−107.38, −9.95)] significantly decreased the triglyceride levels of patients with MetS, as presented in Table 6.

**Table 6:** League table of all pairwise comparisons of the effects of dietary patterns on triglyceride in MetS patients.

TG						
**DASH diet**						
−19.90 (−52.96, 13.16)	**Vegan diet**					
23.50 (−20.21, 67.21)	43.40 (−6.46, 80.35)	**Low-carbohydrate diet**				
−3.95 (−37.39, 29.50)	15.96 (−2.66, 34.57)	−27.45 (−62.96, 8.07)	**Mediterranean diet**			
−2.07 (−40.84, 36.69)	17.83 (−13.11, 48.76)	−25.58 (−45.73, −5.42)	1.87 (−27.33, 31.08)	**Low-fat diet**		
44.92 (−11.06, 100.91)	64.83 (−13.94, 115.71)	21.42 (−23.73, 66.57)	48.87 (−0.98, 98.72)	47.00 (6.60, 87.40)	**Ketogenic diet**	
−13.74 (−41.29, 13.81)	6.16 (−12.28, 24.60)	**−37.24 (−71.13, −3.35)**	−9.80 (−28.92, 9.32)	−11.67 (−38.89, 15.56)	**−58.66 (−107.38, −9.95)**	**Control diet**

Statistically significant treatment effects are in bold.

The results of SUCRA ranking were as follows: the ketogenic diet (SUCRA = 94.9%) > low-carbohydrate diet (SUCRA = 82.6%) > DASH diet (SUCRA = 50.8%) > low-fat diet (SUCRA = 46.1%) > Mediterranean diet (SUCRA = 45.5%) > control diet (SUCRA = 21.0%) > vegan diet (SUCRA = 9.0%).

### Results of network meta-analysis on HDL-C

The network meta-analysis findings revealed that, when compared with the control diet group, no dietary patterns significantly elevated HDL-C levels in patients with MetS, as presented in Table 7.

**Table 7:** League table of all pairwise comparisons of the effects of dietary patterns on HDL-C in MetS patients.

HDL-C						
**DASH diet**						
5.11 (−0.10, 10.33)	**Vegan diet**					
−0.06 (−6.98, 6.86)	−5.17 (−11.69, 1.34)	**Low-carbohydrate diet**				
1.46 (−3.66, 6.58)	−3.65 (−7.36, 0.06)	1.52 (−4.63, 7.68)	**Mediterranean diet**			
1.85 (−3.97, 7.68)	−3.26 (−8.58, 2.06)	1.92 (−1.83, 5.67)	0.39 (−4.51, 5.29)	**Low-fat diet**		
−0.15 (−10.36, 10.06)	−5.26 (−15.19, 4.67)	−0.08 (−9.27, 9.10)	−1.61 (−11.32, 8.10)	−2.00 (−10.39, 6.39)	**Ketogenic diet**	
1.29 (−2.60, 5.18)	−3.82 (−7.32, 0.32)	1.35 (−4.38, 7.09)	−0.17 (−3.50, 3.16)	−0.56 (−4.92, 3.79)	1.44 (−8.01, 10.89)	**Control diet**

Statistically significant treatment effects are in bold.

The results of SUCRA ranking were as follows: the vegan diet (SUCRA = 92.8%) > low-fat diet (SUCRA = 59.5%) > Mediterranean diet (SUCRA = 49.6%) > control diet (SUCRA = 46.8%) > ketogenic diet (SUCRA = 38.1%) > the low-carbohydrate diet (SUCRA = 32.3%) > DASH diet (SUCRA = 30.8%).

### Results of network meta-analysis on FBG

The findings of the network meta-analysis revealed that, compared to the control diet group, the vegan diet [MD = −0.32, 95% CI (−0.54, −0.10)], the low-carbohydrate diet [MD = −0.34, 95% CI (−0.66, −0.02)], and the Mediterranean diet [MD = −0.34, 95% CI (−0.54, −0.14)] significantly regulated FBG in patients with MetS, as presented in Table 8.

**Table 8:** League table of all pairwise comparisons of the effects of dietary patterns on FBG in MetS patients.

FBG					
**DASH diet**					
0.19 (−0.15, 0.52)	**Vegan diet**				
0.20 (−0.20, 0.60)	0.02 (−0.37, 0.40)	**Low-carbohydrate diet**			
0.20 (−0.10, 0.51)	0.02 (−0.18, 0.22)	0.00 (−0.37, 0.37)	**Mediterranean diet**		
0.06 (−0.27, 0.39)	−0.13 (−0.45, 0.19)	−0.14 (−0.36, 0.07)	−0.15 (−0.45, 0.16)	**Low-fat diet**	
−0.14 (−0.37, 0.10)	**−0.32 (−0.54, −0.10)**	**−0.34 (−0.66, −0.02)**	**−0.34 (−0.54, −0.14)**	−0.19 (−0.42, 0.04)	**Control diet**

Statistically significant treatment effects are in bold.

The SUCRA results were ranked as follows: the Mediterranean diet (SUCRA = 75.7%) > low-carbohydrate diet (SUCRA = 75.1%) > vegan diet (SUCRA = 70.6%) > low-fat diet (SUCRA = 42.0%) > DASH diet (SUCRA = 32.5%) > control diet (SUCRA = 4.1%).

### Sensitivity analyses

Sensitivity analyses were further performed for studies with intervention durations ranging from 12 to 48 weeks. The results revealed that the ranking of the relative effects of each dietary pattern on the different outcome measures was generally consistent with the results of the previous overall analysis, after the intervention that was too short or too long was excluded.

Notably, within this time frame, some dietary patterns showed different effects than those in the previous analysis: a low-fat diet showed a statistically significant triglyceride-lowering effect. Meanwhile, the increase of HDL-C level in the DASH diet was also significant. In addition, the SUCRA ranking of FBG indicators was changed, and the low-carbohydrate diet and DASH diet showed better intervention potential. These findings suggest that intervention duration may be an important factor affecting the effects of specific dietary patterns, and more attention should be paid to the intervention duration in future clinical practice and research (4). Specific results are presented in Supplementary File 2.

### Publication bias

**Figure 5:**
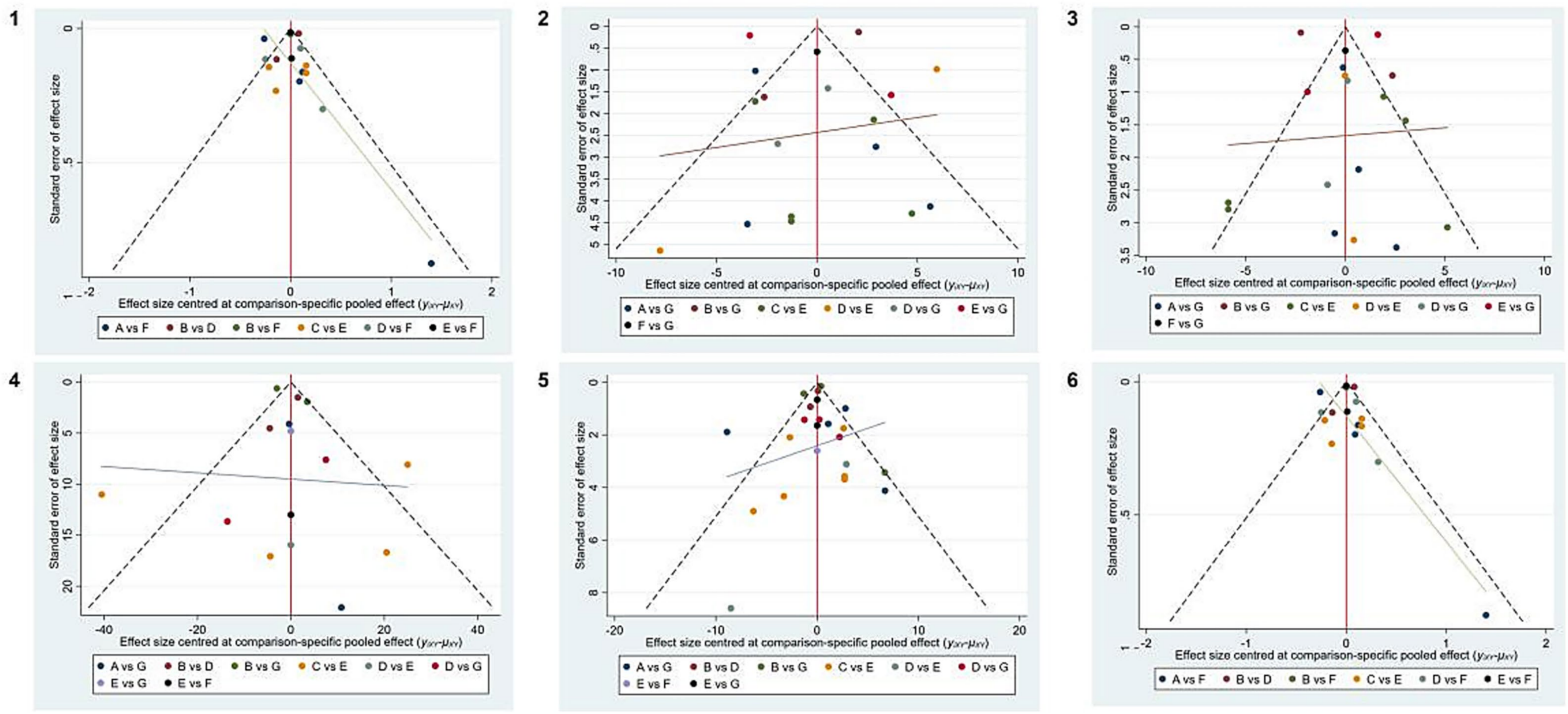
Publication bias of different outcome measures. **1**, WC; **2**, SBP; **3**, DBP; **4**, TG; **5**, HDL-C; **6**, FBG. A, DASH diet; B, vegan diet; C, low-carbohydrate diet; D, Mediterranean diet; E, low-fat diet, F, ketogenic diet, and G, control diet.

Funnel plots were constructed using WC, blood pressure, TG, HDL-C, and FBG as outcome indicators. The results indicated that the funnel plots were essentially symmetrical. However, in all five outcome indicators, some studies fell outside the funnel. There remained a possibility of publication bias within the research network. The specific details of publication bias are shown in Figure 5.

## Discussion

This research carried out a network meta-analysis to indirectly compare the impacts of six dietary patterns on Mets, based on five outcome indicators: WC, blood pressure, triglycerides, high-density lipoprotein cholesterol, and FBG. The objective was to identify more specific dietary patterns and offer evidence-based medical references for clinical practice. A total of 26 RCTs were incorporated into this study. Regarding the reduction of WC and the increase of high-density lipoprotein cholesterol levels, a vegan diet proved to be the most favorable option. With regard to lowering blood pressure and triglyceride levels, the ketogenic diet proved to be the most effective. In terms of controlling FBG, the Mediterranean diet showed the best performance.

The vegetarian diet is particularly effective in reducing waist circumference and increasing high-density lipoprotein cholesterol (HDL-C) levels. In line with previous research, Chen et al. (48) conducted a 7-year follow-up study to assess the potential of a vegetarian diet in preventing abdominal fat accumulation among older adults. The findings indicated that adherence to a vegetarian diet led to reductions in body mass index, as well as waist and hip circumferences, along with decreased fat mass. The underlying mechanism may involve the high dietary fiber content and low saturated fatty acid levels characteristic of vegetarian diets, which can significantly enhance satiety and reduce overall energy intake. Furthermore, this dietary approach may improve insulin sensitivity and lipid metabolism by modulating gut microbiota and promoting the production of short-chain fatty acids (49–51).

The ketogenic diet has demonstrated significant effects in reducing blood pressure and triglyceride levels. A meta-analysis conducted by Zhou et al. (52) indicates that the ketogenic diet can effectively improve weight, blood glucose, and lipid profiles in overweight patients with type 2 diabetes. Another meta-analysis evaluating the potential of a very low-calorie ketogenic diet (VLCKD) in treating obesity showed that VLCKD outperformed other diets in improving multiple parameters, including total cholesterol and triglycerides. Among other things, a significant reduction in LDL cholesterol was observed, but these changes were similar to those seen with other weight loss interventions (53). The ketogenic diet is characterized by a specific macronutrient ratio that induces and maintains a metabolic state known as “ketosis.” The significantly reduced carbohydrate intake leads to decreased insulin levels, which promotes fat mobilization and ketone body production. This process enhances fatty acid oxidation, suppresses *de novo* lipogenesis in the liver, and improves lipoprotein composition (54, 55). However, since certain tissues within the human body are unable to utilize ketone bodies, the ketogenic diet may not be suitable for long-term adherence (56). Therefore, it should be implemented as a short-term intervention under medical supervision.

The results of this study indicate that the Mediterranean diet can significantly improve blood glucose levels, a conclusion consistent with multiple previous meta-analyses. Huo et al. (57) have confirmed through their meta-analysis that the improvement in blood glucose control associated with the Mediterranean diet is more pronounced compared to control diets. Furthermore, Slavin (58) highlighted in their meta-analysis that the primary source of dietary fat in the Mediterranean diet is olive oil, which has been shown to possess health benefits and is linked to a reduced risk of developing type 2 diabetes as well as improved glucose metabolism. This dietary pattern is rich in monounsaturated fatty acids, polyphenols, and dietary fiber. Existing research suggests that these components may support blood glucose regulation by exerting anti-inflammatory and antioxidant effects and enhancing endothelial function and insulin sensitivity (59–63). Additionally, ingredients such as olive oil, nuts, and whole grains within this dietary model contribute to appetite regulation and metabolic health.

Furthermore, we extracted data on adverse events and dropout rates from all included studies to assess adherence to the interventions. Among the 26 studies considered, 24 reported dropout information, accounting for 76.9% of the population. The overall dropout rate varied from 0 to 34%, with one study reporting the highest dropout rate of 34% (28). We utilized the dropout rate as one of the criteria for assessing RoB. In our evaluation of bias risk, a higher dropout rate was a significant factor contributing to some studies being classified as high risk. Additionally, two studies reported adverse events occurring during the intervention period. One study noted that, in the vegetarian group, there was one case of hypoglycemia, and two participants in the intervention group experienced low serum B12 levels; these individuals recovered after supplementation. There was also one case of cholecystectomy unrelated to dietary factors. Another study reported a decrease in vitamin B12 levels and an increase in IL-17 among participants in the vegetarian group (42).

The findings of this study indicate the necessity for the development of individualized, precision nutrition strategies. Clinicians should prioritize specific dietary patterns that address patients’ most significant metabolic abnormalities. The ultimate dietary choice must be customized to align with individual preferences, cultural contexts, and lifestyles to ensure feasibility and long-term sustainability. It is essential to provide ongoing nutrition education, behavioral support, and regular monitoring to assist patients in making informed dietary choices, establishing healthy eating habits, and ultimately achieving sustained compliance. Future research should further investigate how these evidence-based dietary patterns can be effectively integrated into routine clinical practice and public health policy.

### Limitations

This study also has some limitations: (1) most of the studies included in this analysis characterized the control diet as either a “regular diet” or one that adheres to “National Dietary Guidelines.” However, there was often a lack of uniformity and detailed descriptions regarding the macronutrient composition and food items within these control diets. Such discrepancies may contribute to clinical heterogeneity and complicate the interpretation of results; (2) confounding factors could potentially influence the outcomes of this analysis. First, energy restriction serves as a critical influencing factor. Among the 26 studies reviewed, 14 used an energy-restricted design, 8 studies were non-energy-restricted, and 4 studies did not report whether they were energy-restricted. This variability in study design can significantly impact changes observed across various measures of MetS and may confound assessments regarding the independent effects associated with different dietary patterns. Second, participants’ adherence to their prescribed diets represents another crucial factor affecting outcomes. The methodologies for assessing dietary adherence varied among studies; some relied on self-reported food diaries while others utilized objective biomarkers. Self-reporting methods are susceptible to measurement errors and recall bias, which may lead to an overestimation of actual adherence levels.

## Conclusion

In conclusion, existing evidence indicates that vegan, ketogenic, and Mediterranean diets have greater potential for improving multiple metabolic indicators, such as blood glucose, blood lipids, and WC, in patients with metabolic syndrome compared to certain other dietary patterns. Nevertheless, additional high-quality and long-term studies are required to boost the credibility of these findings.

## Data Availability

The original contributions presented in the study are included in the article/Supplementary material, further inquiries can be directed to the corresponding authors.

## Supplementary material

SUPPLEMENTARY FILE 1 The search strategy.

SUPPLEMENTARY FILE 2 Sensitivity analysis.

## References

[1] Sigmund CD Carey RM Appel LJ Arnett DK Bosworth HB Cushman WC . Report of the national heart, lung, and blood institute working group on hypertension: barriers to translation. Hypertension. (2020) 75:902–17. doi: 10.1161/HYPERTENSIONAHA.119.13887, PMID: 32063061 PMC7067675

[2] Bovolini A Garcia J Andrade MA Duarte JA. Metabolic syndrome pathophysiology and predisposing factors. Int J Sports Med. (2021) 42:199–214. doi: 10.1055/a-1263-0898, PMID: 33075830

[3] Garralda-Del-Villar M Carlos-Chillerón S Diaz-Gutierrez J Ruiz-Canela M Gea A Martínez-González MA . Healthy lifestyle and incidence of metabolic syndrome in the SUN cohort. Nutrients. (2018) 11:65. doi: 10.3390/nu11010065, PMID: 30598006 PMC6357191

[4] Gurka MJ Guo Y Filipp SL DeBoer MD. Metabolic syndrome severity is significantly associated with future coronary heart disease in type 2 diabetes. Cardiovasc Diabetol. (2018) 17:17. doi: 10.1186/s12933-017-0647-y, PMID: 29351794 PMC5775549

[5] Prevention, Diabetes, and Treatment of Clinical Guidelines Writing Group. Guidelines for the prevention and treatment of type 2 diabetes in China (2020 edition) (part 1). Chin J Practical Med. (2021) 41:668–95. doi: 10.19538/j.nk2021080106

[6] Fahed G Aoun L Bou Zerdan M Allam S Bou Zerdan M Bouferraa Y . Metabolic syndrome: updates on pathophysiology and management in 2021. Int J Mol Sci. (2022) 23:786. doi: 10.3390/ijms23020786, PMID: 35054972 PMC8775991

[7] Saklayen MG. The global epidemic of the metabolic syndrome. Curr Hypertens Rep. (2018) 20:12. doi: 10.1007/s11906-018-0812-z, PMID: 29480368 PMC5866840

[8] Grundy SM Stone NJ Bailey AL Beam C Birtcher KK Blumenthal RS . 2018 AHA/ACC/AACVPR/AAPA/ABC/ACPM/ADA/AGS/APhA/ASPC/NLA/PCNA guideline on the management of blood cholesterol: a report of the American College of Cardiology/American Heart Association Task Force on clinical practice guidelines. Circulation. (2019) 139:e1082–143. doi: 10.1161/CIR.0000000000000625, PMID: 30586774 PMC7403606

[9] Karr S. Epidemiology and management of hyperlipidemia. Am J Manag Care. (2017) 23:S139–48. 28978219

[10] Di Daniele N Noce A Vidiri MF Moriconi E Marrone G Annicchiarico-Petruzzelli M . Impact of Mediterranean diet on metabolic syndrome, cancer and longevity. Oncotarget. (2017) 8:8947–79. doi: 10.18632/oncotarget.13553, PMID: 27894098 PMC5352455

[11] Tyrovola D Soulaidopoulos S Tsioufis C Lazaros G. The role of nutrition in cardiovascular disease: current concepts and trends. Nutrients. (2023) 15:1064. doi: 10.3390/nu15051064, PMID: 36904064 PMC10005442

[12] Yang D Caiying F Jing L Jie Y Wenyi L Yuzhu X. (2022). Meta-analysis of the Relationship between Ketogenic Diet and Metabolic Syndrome and its Influencing Factors. Chinese Nutrition Society. 525. doi: 10.26914/c.cnkihy.2022.034057

[13] Valenzuela-Fuenzalida JJ Bravo VS Valarezo LM Delgado Retamal MF Leiva JM Bruna-Mejías A . Effectiveness of DASH diet versus other diet modalities in patients with metabolic syndrome: a systematic review and meta-analysis. Nutrients. (2024) 16:3054. doi: 10.3390/nu16183054, PMID: 39339654 PMC11434995

[14] Picasso MC Lo-Tayraco JA Ramos-Villanueva JM Pasupuleti V Hernandez AV. Effect of vegetarian diets on the presentation of metabolic syndrome or its components: a systematic review and meta-analysis. Clin Nutr. (2019) 38:1117–32. doi: 10.1016/j.clnu.2018.05.021, PMID: 29907356

[15] Bakaloudi DR Chrysoula L Kotzakioulafi E Theodoridis X Chourdakis M. Impact of the level of adherence to Mediterranean diet on the parameters of metabolic syndrome: a systematic review and meta-analysis of observational studies. Nutrients. (2021) 13:1514. doi: 10.3390/nu13051514, PMID: 33946280 PMC8146502

[16] Willems AEM Sura-de Jong M van Beek AP Nederhof E van Dijk G. Effects of macronutrient intake in obesity: a meta-analysis of low-carbohydrate and low-fat diets on markers of the metabolic syndrome. Nutr Rev. (2021) 79:429–44. doi: 10.1093/nutrit/nuaa044, PMID: 32885229 PMC7947787

[17] Page MJ McKenzie JE Bossuyt PM Boutron I Hoffmann TC Mulrow CD . The PRISMA 2020 statement: an updated guideline for reporting systematic reviews. BMJ. (2021) 372:n71. doi: 10.1136/bmj.n71 33782057 PMC8005924

[18] Castro-Barquero S Ruiz-León AM Sierra-Pérez M Estruch R Casas R. Dietary strategies for metabolic syndrome: a comprehensive review. Nutrients. (2020) 12:2983. doi: 10.3390/nu12102983, PMID: 33003472 PMC7600579

[19] Haider LM Schwingshackl L Hoffmann G Ekmekcioglu C. The effect of vegetarian diets on iron status in adults: a systematic review and meta-analysis. Crit Rev Food Sci Nutr. (2018) 58:1359–74. doi: 10.1080/10408398.2016.1259210, PMID: 27880062

[20] Choi YJ Jeon SM Shin S. Impact of a ketogenic diet on metabolic parameters in patients with obesity or overweight and with or without type 2 diabetes: a meta-analysis of randomized controlled trials. Nutrients. (2020) 12:2005. doi: 10.3390/nu12072005, PMID: 32640608 PMC7400909

[21] Sterne JAC Savović J Page MJ Elbers RG Blencowe NS Boutron I . RoB 2: a revised tool for assessing risk of bias in randomised trials. BMJ. (2019) 366:l4898. doi: 10.1136/bmj.l4898, PMID: 31462531

[22] Choi SH Choi-Kwon S. The effects of the DASH diet education program with omega-3 fatty acid supplementation on metabolic syndrome parameters in elderly women with abdominal obesity. Nutr Res Pract. (2015) 9:150–7. doi: 10.4162/nrp.2015.9.2.150, PMID: 25861421 PMC4388946

[23] Al-Solaiman Y Jesri A Mountford WK Lackland DT Zhao Y Egan BM. DASH lowers blood pressure in obese hypertensives beyond potassium, magnesium and fibre. J Hum Hypertens. (2010) 24:237–46. doi: 10.1038/jhh.2009.58, PMID: 19626043 PMC2841705

[24] Azadbakht L Mirmiran P Esmaillzadeh A Azizi T Azizi F. Beneficial effects of a dietary approaches to stop hypertension eating plan on features of the metabolic syndrome. Diabetes Care. (2005) 28:2823–31. doi: 10.2337/diacare.28.12.2823, PMID: 16306540

[25] Kucharska A Gajewska D Kiedrowski M Sińska B Juszczyk G Czerw A . The impact of individualised nutritional therapy according to DASH diet on blood pressure, body mass, and selected biochemical parameters in overweight/obese patients with primary arterial hypertension: a prospective randomised study. Kardiol Pol. (2018) 76:158–65. doi: 10.5603/KP.a2017.0184, PMID: 28980293

[26] Paula TP Viana LV Neto ATZ Leitão CB Gross JL Azevedo MJ. Effects of the DASH diet and walking on blood pressure in patients with type 2 diabetes and uncontrolled hypertension: a randomized controlled trial. J Clin Hypertens. (2015) 17:895–901. doi: 10.1111/jch.12597, PMID: 26041459 PMC8031764

[27] Wright N Wilson L Smith M Duncan B McHugh P. The BROAD study: a randomised controlled trial using a whole food plant-based diet in the community for obesity, ischaemic heart disease or diabetes. Nutr Diabetes. (2017) 7:e256. doi: 10.1038/nutd.2017.3, PMID: 28319109 PMC5380896

[28] Mishra S Xu J Agarwal U Gonzales J Levin S Barnard ND. A multicenter randomized controlled trial of a plant-based nutrition program to reduce body weight and cardiovascular risk in the corporate setting: the GEICO study. Eur J Clin Nutr. (2013) 67:718–24. doi: 10.1038/ejcn.2013.92, PMID: 23695207 PMC3701293

[29] Barnard ND Alwarith J Rembert E Brandon L Nguyen M Goergen A . A Mediterranean diet and low-fat vegan diet to improve body weight and cardiometabolic risk factors: a randomized, cross-over trial. J Am Nutr Assoc. (2022) 41:127–39. doi: 10.1080/07315724.2020.1869625, PMID: 33544066

[30] Kahleova H Petersen KF Shulman GI Alwarith J Rembert E Tura A . Effect of a low-fat vegan diet on body weight, insulin sensitivity, postprandial metabolism, and intramyocellular and hepatocellular lipid levels in overweight adults: a randomized clinical trial. JAMA Netw Open. (2020) 3:e2025454. doi: 10.1001/jamanetworkopen.2020.25454, PMID: 33252690 PMC7705596

[31] Volek JS Phinney SD Forsythe CE Quann EE Wood RJ Puglisi MJ . Carbohydrate restriction has a more favorable impact on the metabolic syndrome than a low fat diet. Lipids. (2009) 44:297–309. doi: 10.1007/s11745-008-3274-2, PMID: 19082851

[32] Bradley U Spence M Courtney CH McKinley MC Ennis CN McCance DR . Low-fat versus low-carbohydrate weight reduction diets: effects on weight loss, insulin resistance, and cardiovascular risk: a randomized control trial. Diabetes. (2009) 58:2741–8. doi: 10.2337/db09-0098, PMID: 19720791 PMC2780863

[33] Volek JS Sharman MJ Gómez AL DiPasquale C Roti M Pumerantz A . Comparison of a very low-carbohydrate and low-fat diet on fasting lipids, LDL subclasses, insulin resistance, and postprandial lipemic responses in overweight women. J Am Coll Nutr. (2004) 23:177–84. doi: 10.1080/07315724.2004.10719359, PMID: 15047685

[34] Pinsawas B Surawit A Mongkolsucharitkul P Pongkunakorn T Suta S Manosan T . Asian low-carbohydrate diet with increased whole egg consumption improves metabolic outcomes in metabolic syndrome: a 52-week intervention study. J Nutr. (2024) 154:3331–45. doi: 10.1016/j.tjnut.2024.08.027, PMID: 39245182

[35] Saslow LR Daubenmier JJ Moskowitz JT Kim S Murphy EJ Phinney SD . Twelve-month outcomes of a randomized trial of a moderate-carbohydrate versus very low-carbohydrate diet in overweight adults with type 2 diabetes mellitus or prediabetes. Nutr Diabetes. (2017) 7:304. doi: 10.1038/s41387-017-0006-9, PMID: 29269731 PMC5865541

[36] Veum VL Laupsa-Borge J Eng Ø Rostrup E Larsen TH Nordrehaug JE . Visceral adiposity and metabolic syndrome after very high-fat and low-fat isocaloric diets: a randomized controlled trial. Am J Clin Nutr. (2017) 105:85–99. doi: 10.3945/ajcn.115.123463, PMID: 27903520

[37] Esposito K Marfella R Ciotola M Di Palo C Giugliano F Giugliano G . Effect of a Mediterranean-style diet on endothelial dysfunction and markers of vascular inflammation in the metabolic syndrome: a randomized trial. JAMA. (2004) 292:1440–6. doi: 10.1001/jama.292.12.1440, PMID: 15383514

[38] Elhayany A Lustman A Abel R Attal-Singer J Vinker S. A low carbohydrate Mediterranean diet improves cardiovascular risk factors and diabetes control among overweight patients with type 2 diabetes mellitus: a 1-year prospective randomized intervention study. Diabetes Obes Metab. (2010) 12:204–9. doi: 10.1111/j.1463-1326.2009.01151.x, PMID: 20151996

[39] Richard C Couture P Desroches S Charest A Lamarche B. Effect of the Mediterranean diet with and without weight loss on cardiovascular risk factors in men with the metabolic syndrome. Nutr Metab Cardiovasc Dis. (2011) 21:628–35. doi: 10.1016/j.numecd.2010.01.012, PMID: 20554173

[40] Fortin A Rabasa-Lhoret R Lemieux S Labonté ME Gingras V. Comparison of a Mediterranean to a low-fat diet intervention in adults with type 1 diabetes and metabolic syndrome: a 6-month randomized trial. Nutr Metab Cardiovasc Dis. (2018) 28:1275–84. doi: 10.1016/j.numecd.2018.08.005 30459054

[41] Wade AT Davis CR Dyer KA Hodgson JM Woodman RJ Murphy KJ. A Mediterranean diet supplemented with dairy foods improves markers of cardiovascular risk: results from the MedDairy randomized controlled trial. Am J Clin Nutr. (2018) 108:1166–82. doi: 10.1093/ajcn/nqy207, PMID: 30351388

[42] Sofi F Dinu M Pagliai G Cesari F Gori AM Sereni A . Low-calorie vegetarian versus Mediterranean diets for reducing body weight and improving cardiovascular risk profile: CARDIVEG study (cardiovascular prevention with vegetarian diet). Circulation. (2018) 137:1103. doi: 10.1161/CIRCULATIONAHA.117.030088, PMID: 29483085

[43] Tierney AC McMonagle J Shaw DI Gulseth HL Helal O Saris WHM . Effects of dietary fat modification on insulin sensitivity and on other risk factors of the metabolic syndrome—LIPGENE: a European randomized dietary intervention study. Int J Obes. (2011) 35:800–9. doi: 10.1038/ijo.2010.209, PMID: 20938439

[44] Meneses ME Camargo A Perez-Martinez P Delgado-Lista J Cruz-Teno C Jimenez-Gomez Y . Postprandial inflammatory response in adipose tissue of patients with metabolic syndrome after the intake of different dietary models. Mol Nutr Food Res. (2011) 55:1759–70. doi: 10.1002/mnfr.201100200, PMID: 22144044

[45] Cruz-Teno C Pérez-Martínez P Delgado-Lista J Yubero-Serrano EM García-Ríos A Marín C . Dietary fat modifies the postprandial inflammatory state in subjects with metabolic syndrome: the LIPGENE study. Mol Nutr Food Res. (2012) 56:854–65. doi: 10.1002/mnfr.201200096, PMID: 22707261

[46] Camhi SM Stefanick ML Katzmarzyk PT Young DR. Metabolic syndrome and changes in body fat from a low-fat diet and/or exercise randomized controlled trial. Obesity. (2010) 18:548–54. doi: 10.1038/oby.2009.304, PMID: 19798074 PMC4279708

[47] Fernandez CA Potts K Bazzano LA. Effect of ideal protein versus low-fat diet for weight loss: a randomized controlled trial. Obes Sci Pract. (2022) 8:299–307. doi: 10.1002/osp4.567, PMID: 35664249 PMC9159558

[48] Chen Z Schoufour JD Rivadeneira F Lamballais S Ikram MA Franco OH . Plant-based diet and adiposity over time in a middle-aged and elderly population: the Rotterdam study. Epidemiology. (2019) 30:303–10. doi: 10.1097/EDE.0000000000000961, PMID: 30507650

[49] Dhingra D Michael M Rajput H Patil RT. Dietary fibre in foods: a review. J Food Sci Technol. (2012) 49:255–66. doi: 10.1007/s13197-011-0365-5, PMID: 23729846 PMC3614039

[50] Hervik AK Svihus B. The role of fiber in energy balance. J Nutr Metab. (2019) 2019:4983657. doi: 10.1155/2019/4983657, PMID: 30805214 PMC6360548

[51] Trautwein EA McKay S. The role of specific components of a plant-based diet in management of dyslipidemia and the impact on cardiovascular risk. Nutrients. (2020) 12:2671. doi: 10.3390/nu12092671, PMID: 32883047 PMC7551487

[52] Zhou C Wang M Liang J He G Chen N. Ketogenic diet benefits to weight loss, glycemic control, and lipid profiles in overweight patients with type 2 diabetes mellitus: a meta-analysis of randomized controlled trails. Int J Environ Res Public Health. (2022) 19:10429. doi: 10.3390/ijerph191610429, PMID: 36012064 PMC9408028

[53] Muscogiuri G El Ghoch M Colao A Hassapidou M Yumuk V Busetto L . European guidelines for obesity management in adults with a very low-calorie ketogenic diet: a systematic review and meta-analysis. Obes Facts. (2021) 14:222–45. doi: 10.1159/000515381, PMID: 33882506 PMC8138199

[54] Volek JS Feinman RD. Carbohydrate restriction improves the features of metabolic syndrome. Metabolic syndrome may be defined by the response to carbohydrate restriction. Nutr Metab. (2005) 2:31. doi: 10.1186/1743-7075-2-31, PMID: 16288655 PMC1323303

[55] Bueno NB de Melo IS de Oliveira SL da Rocha Ataide T. Very-low-carbohydrate ketogenic diet vs. low-fat diet for long-term weight loss: a meta-analysis of randomised controlled trials. Br J Nutr. (2013) 110:1178–87. doi: 10.1017/S0007114513000548 23651522

[56] Kolb H Kempf K Röhling M Lenzen-Schulte M Schloot NC Martin S. Ketone bodies: from enemy to friend and guardian angel. BMC Med. (2021) 19:313. doi: 10.1186/s12916-021-02185-0, PMID: 34879839 PMC8656040

[57] Huo R Du T Xu Y Xu W Chen X Sun K . Effects of Mediterranean-style diet on glycemic control, weight loss and cardiovascular risk factors among type 2 diabetes individuals: a meta-analysis. Eur J Clin Nutr. (2015) 69:1200–8. doi: 10.1038/ejcn.2014.243, PMID: 25369829

[58] Slavin JL. Dietary fiber and body weight. Nutrition. (2005) 21:411–8. doi: 10.1016/j.nut.2004.08.018, PMID: 15797686

[59] Rehman K Haider K Jabeen K Akash MSH. Current perspectives of oleic acid: regulation of molecular pathways in mitochondrial and endothelial functioning against insulin resistance and diabetes. Rev Endocr Metab Disord. (2020) 21:631–43. doi: 10.1007/s11154-020-09549-6, PMID: 32125563

[60] Santos-Buelga C González-Manzano S González-Paramás AM. Wine, polyphenols, and Mediterranean diets. What else is there to say? Molecules. (2021) 26:5537. doi: 10.3390/molecules26185537, PMID: 34577008 PMC8468969

[61] Cannataro R Fazio A La Torre C Caroleo MC Cione E. Polyphenols in the Mediterranean diet: from dietary sources to microRNA modulation. Antioxidants. (2021) 10:328. doi: 10.3390/antiox10020328, PMID: 33672251 PMC7926722

[62] Giroli MG Werba JP Risé P Porro B Sala A Amato M . Effects of Mediterranean diet or low-fat diet on blood fatty acids in patients with coronary heart disease. A randomized intervention study. Nutrients. (2021) 13:2389. doi: 10.3390/nu13072389, PMID: 34371898 PMC8308706

[63] Schwingshackl L Morze J Hoffmann G. Mediterranean diet and health status: active ingredients and pharmacological mechanisms. Br J Pharmacol. (2020) 177:1241–57. doi: 10.1111/bph.14778, PMID: 31243760 PMC7056467

