# Supplementary material for: Network meta-analysis of the effects of different dietary patterns on patients with metabolic syndrome
Source: Front Nutr. 2025 Oct 22;12:1634545. doi: 10.3389/fnut.2025.1634545 (PMC12585985; doi:10.3389/fnut.2025.1634545)
Supplement: Supplementary file 1 [file Table_1.docx]

| PubMed | | |
| --- | --- | --- |
| #1 | (metabolic syndrome[Title/Abstract])OR (Metabolic Syndrome[MeSH Terms]) | 77141 |
| #2 | (((((((((((((((((((((((((((diet*[Title/Abstract]) OR (dietary pattern*[Title/Abstract])) OR (Diet, Ketogenic[MeSH Terms])) OR (Ketogenic diet*[Title/Abstract])) OR (Keto diet*[Title/Abstract])) OR (Diet, DASH[MeSH Terms])) OR (DASH diet*[Title/Abstract])) OR (Dietary Approaches to Stop Hypertension[Title/Abstract])) OR (DASH eating plan[Title/Abstract])) OR (Diet, Vegetarian[MeSH Terms])) OR (Vegetarian diet*[Title/Abstract])) OR (Vegan diet*[Title/Abstract])) OR (plant-based diet*[Title/Abstract])) OR (meat-free diet*[Title/Abstract])) OR (Diet, Mediterranean[MeSH Terms])) OR (Mediterranean diet*[Title/Abstract])) OR (Mediterranean dietary pattern[Title/Abstract])) OR (MedDiet[Title/Abstract])) OR (cretan diet[Title/Abstract])) OR (Diet, Fat-Restricted[MeSH Terms])) OR (Low-fat diet*[Title/Abstract])) OR (fat-restricted diet*[Title/Abstract])) OR (reduced-fat diet*[Title/Abstract])) OR (hypolipidemic diet*[Title/Abstract])) OR (Diet, Carbohydrate-Restricted[MeSH Terms])) OR (Low-carbohydrate diet*[Title/Abstract])) OR (low-carb diet*[Title/Abstract])) OR (carb-restricted diet*[Title/Abstract]) | 759947 |
| #3 | ((((Randomized Controlled Trials[Publication Type]) OR (controlled clinical trial[Publication Type])) OR (randomized[Title/Abstract])) OR (randomly[Title/Abstract])) | 1390090 |
| #4 | #1+#2+#3 | 1903 |

| Embase | | |
| --- | --- | --- |
| #1 | 'metabolic syndrome'/exp OR 'metabolic syndrome':ab,ti OR 'metabolic syndromes':ab,ti OR 'mets':ab,ti | 142329 |
| #2 | 'ketogenic diet'/exp OR 'ketogenic diet':ab,ti OR 'ketogenic diets':ab,ti OR 'DASH diet'/exp OR 'DASH diet':ab,ti OR 'Dietary Approaches to Stop Hypertension':ab,ti OR 'vegetarian diet'/exp OR 'vegetarian diet':ab,ti OR 'vegetarian diets':ab,ti OR 'mediterranean diet'/exp OR 'mediterranean diet':ab,ti OR 'mediterranean diets':ab,ti OR 'low fat diet'/exp OR 'low fat diet':ab,ti OR 'low fat diets':ab,ti OR 'low-fat diet':ab,ti OR 'low-fat diets':ab,ti OR 'low carbohydrate diet'/exp OR 'low carbohydrate diet':ab,ti OR 'low carbohydrate diets':ab,ti OR 'low-carbohydrate diet':ab,ti OR 'low-carbohydrate diets':ab,ti | 54873 |
| #3 | 'randomized controlled trials':de OR 'controlled clinical trial':de OR 'randomized':ab,ti OR 'randomly':ab,ti | 1828055 |
| #4 | #1+#2+#3 | 612 |

| Cochrane Library | | |
| --- | --- | --- |
| #1 | (metabolic syndrome):ti,ab,kw | 18285 |
| #2 | (diet OR Ketogenic diet OR DASH diet OR Dietary approaches to stop hypertension OR Vegetarian diets OR Mediterranean diet OR Low-fat diets OR Low-carbohydrate diets):ti,ab,kw | 82982 |
| #3 | (metabolic syndrome OR randomized controlled trial OR controlled clinical trial OR randomized OR randomly):ti,ab,kw | 1454376 |
| #4 | #1+#2+#3 | 3834 |

| Web of Science | | |
| --- | --- | --- |
| #1 | (TI=("metabolic syndrome" OR "metabolic syndromes" OR "mets") OR  AB=("metabolic syndrome" OR "metabolic syndromes" OR "mets")) | 80788 |
| #2 | TI=("ketogenic diet" OR "ketogenic diets" OR "DASH diet" OR "Dietary Approaches to Stop Hypertension" OR "vegetarian diet" OR "vegetarian diets" OR "mediterranean diet" OR "mediterranean diets" OR "low fat diet" OR "low fat diets" OR "low-fat diet" OR "low-fat diets" OR"low carbohydrate diet" OR "low carbohydrate diets" OR "low-carbohydrate diet" OR "low-carbohydrate diets") OR AB=("ketogenic diet" OR "ketogenic diets" OR "DASH diet" OR "Dietary Approaches to Stop Hypertension" OR "vegetarian diet" OR "vegetarian diets" OR "mediterranean diet" OR "mediterranean diets" OR "low fat diet" OR "low fat diets" OR "low-fat diet" OR "low-fat diets" OR "low carbohydrate diet" OR "low carbohydrate diets" OR "low-carbohydrate diet" OR "low-carbohydrate diets")) | 26172 |
| #3 | TI=("randomized controlled trial*" OR "randomised controlled trial*" OR "RCT*" OR "controlled clinical trial*" OR "randomized" OR "randomly") OR AB=("randomized controlled trial*" OR "randomised controlled trial*" OR "RCT*" OR "controlled clinical trial*" OR "randomized" OR "randomly")) | 1402952 |
| #4 | #1+#2+#3 | 224 |
