# Supplementary material for: Network meta-analysis of the effects of different dietary patterns on patients with metabolic syndrome
Source: Front Nutr. 2025 Oct 22;12:1634545. doi: 10.3389/fnut.2025.1634545 (PMC12585985; doi:10.3389/fnut.2025.1634545)
Supplement: Supplementary file 2 [file Table_2.docx]

**1.WC**

The findings of the network meta-analysis revealed that, in comparison to the control diet group, the DASH diet [MD = -8.64, 95% CI (-14.07, -3.22)] and the vegan diet [MD = -12.00, 95% CI (-18.62, -5.39)] significantly decreased the waist circumference of patients with MetS.

Table1 League table of all pairwise comparisons of the effects of dietary patterns on WC in MetS patients

(intervention duration: 12 - 48 weeks)

| WC | | | | | | |
| --- | --- | --- | --- | --- | --- | --- |
| **DASH diet** |  |  |  |  |  |  |
| 3.36  (-5.20,11.91) | **Vegan diet** |  |  |  |  |  |
| -6.08  (-15.51,3.36) | -9.44  (-19.65,0.77) | **Low-carbohydrate diet** |  |  |  |  |
| **-7.29**  **(-14.06,-0.51)** | **-10.64**  **(-18.39,-2.90)** | -1.21  (-9.42,7.00) | **Mediterranean diet** |  |  |  |
| -7.48  (-15.11,0.15) | **-10.84**  **(-19.42,-2.25)** | -1.40  (-7.01,4.20) | -0.19  (-6.29,5.90) | **Low-fat diet** |  |  |
| -6.43  (-14.75,1.89) | **-9.79 (-18.94,-0.64)** | -0.35  (-10.37,9.67) | 0.86  (-6.64,8.35) | 1.05  (-7.31,9.41) | **Ketogenic diet** |  |
| **-8.64**  **(-14.07,-3.22)** | **-12.00**  **(-18.62,-5.39)** | -2.56  (-10.34,5.21) | -1.36  (-5.38,2.67) | -1.16  (-6.64,4.31) | -2.21  (-8.53,4.11) | **Control diet** |

The SUCRA ranking results for studies with intervention durations of 12–48 weeks were as follows: vegan diet (SUCRA = 95.1%) > DASH diet (SUCRA = 82.8%) > low-carbohydrate diet (SUCRA = 46.3%) > ketogenic diet (SUCRA = 41.4%) > Mediterranean diet (SUCRA = 34.1%) > low-fat diet (SUCRA = 32.4%) > control diet (SUCRA = 17.9%). The ranking outcomes for each dietary pattern are presented in Figure 1.


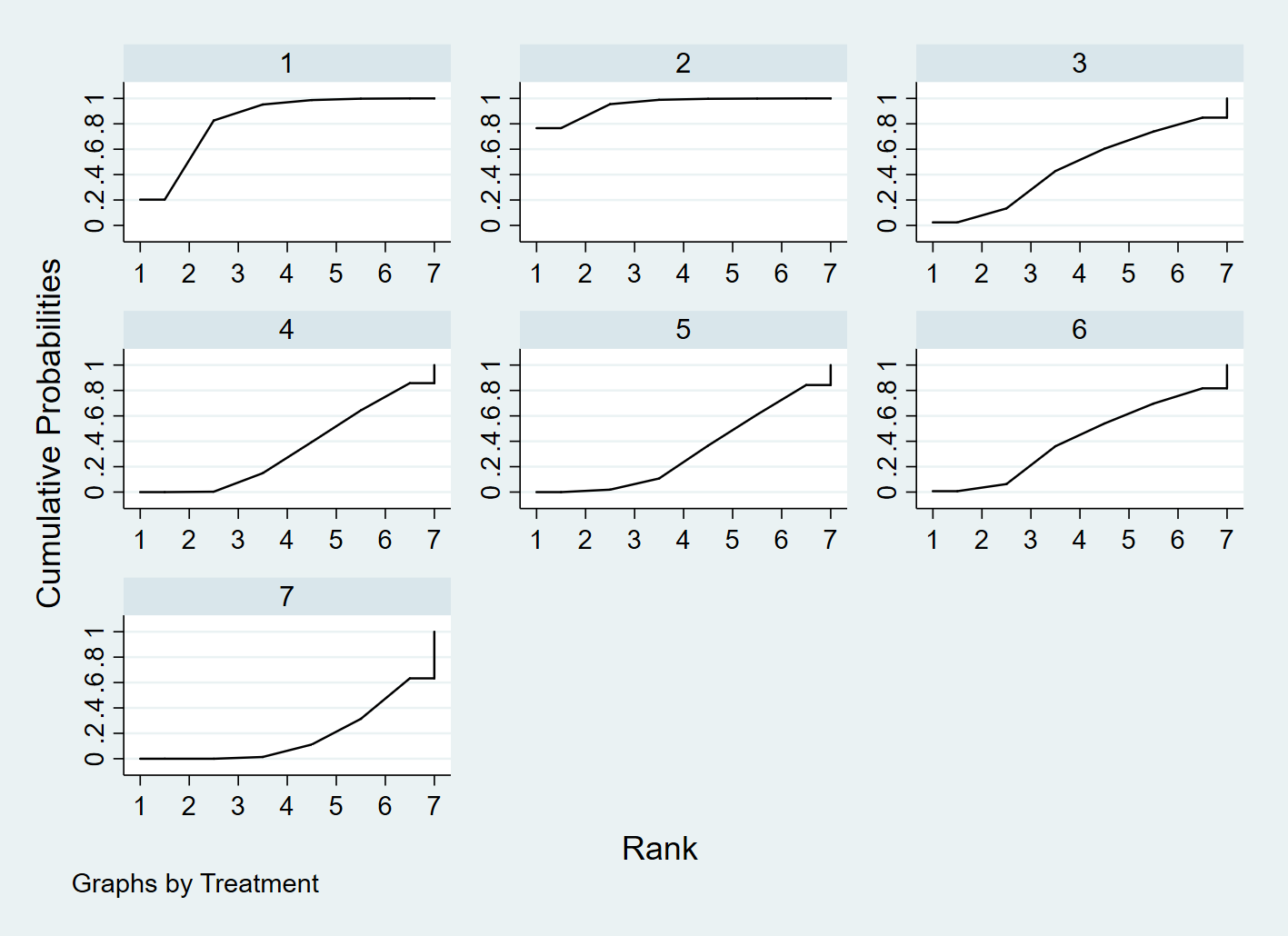


Figure 1 SUCRA plots of WC in MetS patients treated with different dietary patterns

(intervention duration: 12 - 48 weeks)

**2.SBP**

The findings of the network meta-analysis revealed that, in comparison with the control diet group, the DASH diet [MD = -5.44, 95% CI (-11.9, -0.40)] and the ketogenic diet [MD = -11.00, 95% CI (-18.39, -3.61)] significantly decreased the systolic blood pressure of patients with Mets.

Table 2 League table of all pairwise comparisons of the effects of dietary patterns on systolic blood pressure in MetS patients(intervention duration: 12 - 48 weeks)

| SBP | | | | | | |
| --- | --- | --- | --- | --- | --- | --- |
| **DASH diet** |  |  |  |  |  |  |
| -7.82  (-15.74,0.09) | **vegan diet** |  |  |  |  |  |
| -1.10  (-10.12,7.93) | 6.73  (-2.13,15.59) | **Low-carbohydrate diet** |  |  |  |  |
| -6.63  (-16.17,2.91) | 1.19  (-8.06,10.44) | -5.54  (-15.00,3.93) | **Mediterranean diet** |  |  |  |
| -0.76  (-8.46,6.94) | 7.06  (-0.45,14.58) | 0.34  (-4.37,5.04) | 5.87  (-2.34,14.09) | **Low-fat diet** |  |  |
| 5.60  (-3.78,14.99) | 13.43 (4.28,22.57) | 6.70  (-3.48,16.88) | 12.24  (1.68,22.79) | 6.36  (-2.67,15.40) | **Ketogenic diet** |  |
| **-5.40**  **(-11.19,-0.40)** | 2.43  (-2.96,7.81) | -4.30  (-11.30,2.70) | 1.24  (-6.30,8.77) | -4.64  (-9.83,0.56) | **-11.00 (-18.39,-3.61)** | **Control diet** |

The SUCRA ranking results for studies with intervention durations of 12–48 weeks were as follows: ketogenic diet (SUCRA = 94.1%) > DASH diet (SUCRA = 68.3%) > low-carbohydrate diet (SUCRA = 62.1%) > low-fat diet (SUCRA = 65.9%)> control diet (SUCRA = 27.1%) > Mediterranean diet (SUCRA =20.7%) > vegan diet (SUCRA = 11.7%). The ranking outcomes of each dietary pattern are presented in Figure 3.


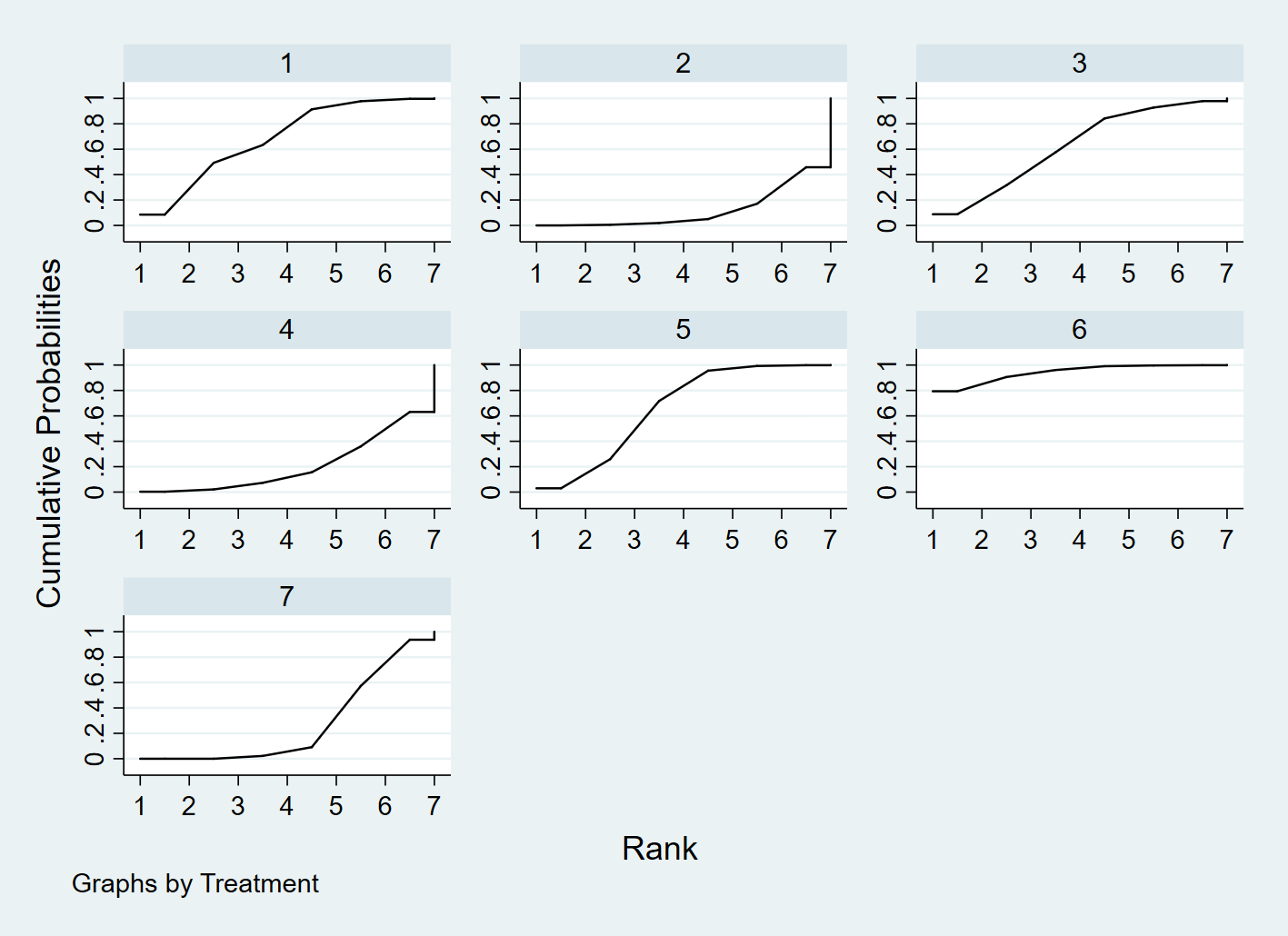


Figure 3 SUCRA plots of SBP in MetS patients treated with different dietary patterns(intervention duration: 12 - 48 weeks)

**3.DBP**

The results of the network meta-analysis showed that compared with the control diet group, the ketogenic diet [MD=-9.39, 95%CI (-15.39, -3.40)] significantly reduced diastolic blood pressure values in patients with MetS, as shown in Table 3.

Table 3 League table of all pairwise comparisons of the effects of dietary patterns on diastolic blood pressure in MetS patients(intervention duration: 12 - 48 weeks)

| DBP | | | | | | |
| --- | --- | --- | --- | --- | --- | --- |
| **DASH diet** |  |  |  |  |  |  |
| -1.47  (-7.99,5.06) | **vegan diet** |  |  |  |  |  |
| -4.13  (-11.52,3.26) | -2.66  (-9.59,4.27) | **Low-carbohydrate diet** |  |  |  |  |
| -1.57  (-9.36,6.21) | -0.11  (-7.47,7.25) | 2.55(-4.62,9.73) | **Mediterranean diet** |  |  |  |
| -4.19  (-10.57,2.20) | -2.72  (-8.60,3.16) | -0.06(-3.71,3.59) | -2.61  (-8.80,3.57) | **Low-fat diet** |  |  |
| 6.28  (-1.49,14.06) | 7.75  (0.39,15.11) | 10.41(2.32,18.51) | 7.86  (-0.62,16.34) | 10.47  (3.24,17.70) | **Ketogenic diet** |  |
| -3.11  (-8.07,1.85) | -1.64  (-5.91,2.63) | 1.02(-4.43,6.46) | -1.53  (-7.53,4.46) | 1.08  (-2.96,5.12) | **-9.39**  **(-15.39,-3.40)** | **Control diet** |

The SUCRA ranking results for studies with intervention durations of 12–48 weeks were as follows: ketogenic diet (SUCRA = 97.9%) > DASH diet (SUCRA =66.6%) > Mediterranean diet (SUCRA = 52.3%) > vegan diet (SUCRA = 53.3%) > control diet (SUCRA = 31.8%) > low-carbohydrate diet (SUCRA = 26.5%) > low-fat diet (SUCRA = 21.6%). The ranking outcomes of each dietary pattern are presented in Figure 3.


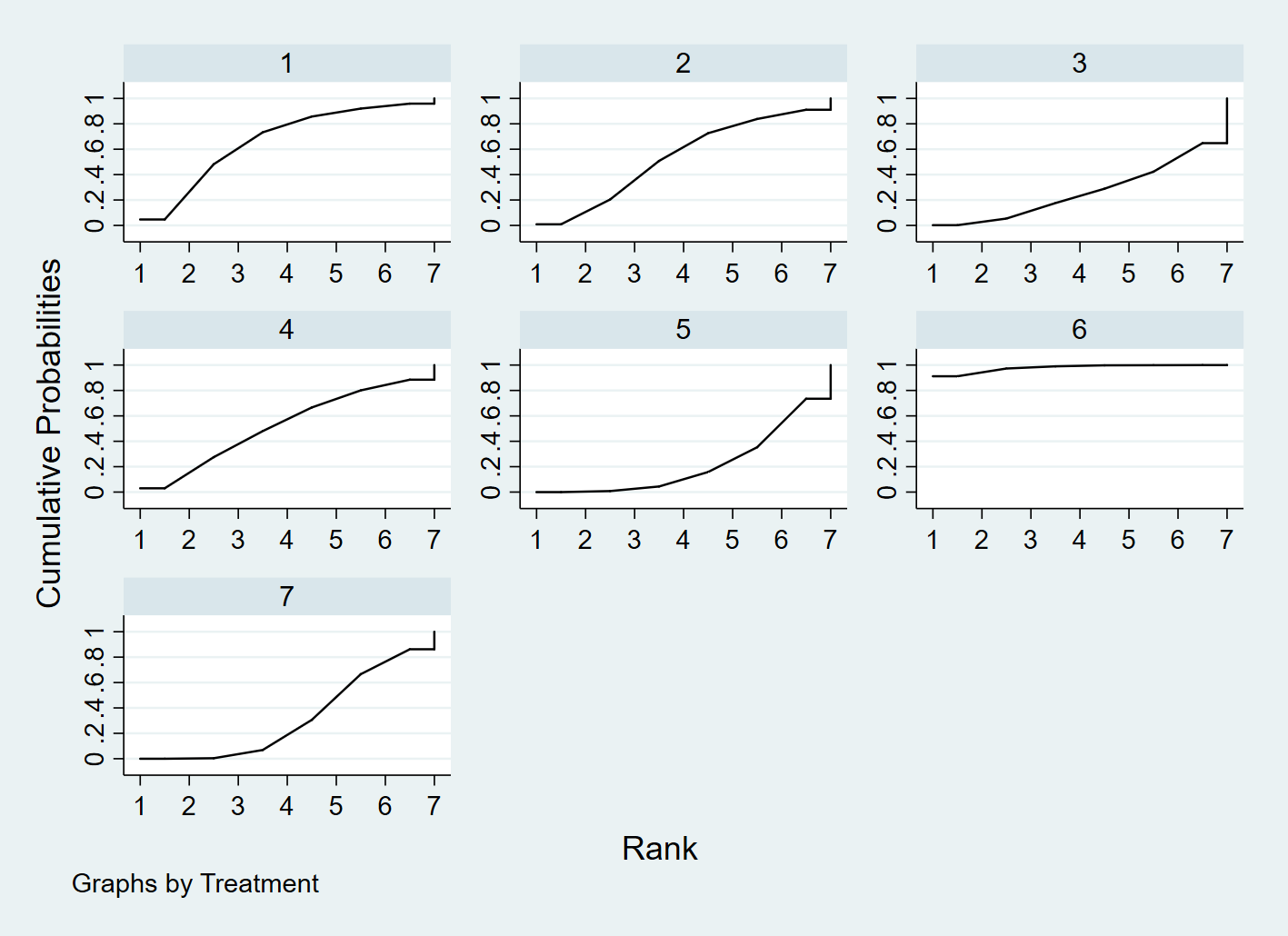


Figure 3 SUCRA plots of DBP in MetS patients treated with different dietary patterns(intervention duration: 12 - 48 weeks)

**4.TG**

The findings of the network meta-analysis indicated that, when compared with the control diet group, the low-carbohydrate diet [MD = -57.96, 95% CI (-79.22, -36.69)], the ketogenic diet [MD = -60.94, 95% CI (-91.00, -30.87)] and Low-fat diet[MD = -13.94, 95% CI (-26.76, -1.12)] significantly decreased the triglyceride levels of patients with MetS, as presented in Table 4.

Table 4 League table of all pairwise comparisons of the effects of dietary patterns on triglyceride in MetS patients(intervention duration: 12 - 48 weeks)

| TG | | | | | | |
| --- | --- | --- | --- | --- | --- | --- |
| **DASH diet** |  |  |  |  |  |  |
| -32.53  (-76.71,11.65) | **Vegan diet** |  |  |  |  |  |
| 36.62  (-11.87,85.12) | 69.15 (-46.33,91.97) | **Low-carbohydrate diet** |  |  |  |  |
| -21.91  (-66.92,23.10) | 10.62 (-2.43,18.81) | -58.53  (-83.36,-33.70) | **Mediterranean diet** |  |  |  |
| -7.40  (-52.83,38.04) | 25.13 (-10.31,39.96) | -44.02  (-60.54,-27.50) | 14.51  (-2.78,31.80) | **Low-fat diet** |  |  |
| 39.60  (-13.35,92.55) | 72.13 (-41.16,103.10) | 2.98  (-28.84,34.80) | 61.51 (29.29,93.74) | 47.00 (19.80,74.20) | **Ketogenic diet** |  |
| -21.34  (-64.95,22.27) | 11.19 (-4.01,18.37) | **-57.96**  **(-79.22,-36.69)** | 0.57  (-10.63,11.77) | **-13.94 (-26.76,-1.12)** | **-60.94 (-91.00,-30.87)** | **Control diet** |

The SUCRA ranking results for studies with intervention durations of 12–48 weeks were as follows: ketogenic diet (SUCRA = 92.0%) > low-carbohydrate diet (SUCRA =89.2%) > DASH diet (SUCRA = 55.8%) > low-fat diet (SUCRA = 55.4%) > Mediterranean diet (SUCRA = 28.5%) > control diet (SUCRA =28.0%) > vegan diet (SUCRA = 1.3%). The ranking outcomes of each dietary pattern are presented in Figure 4.

**
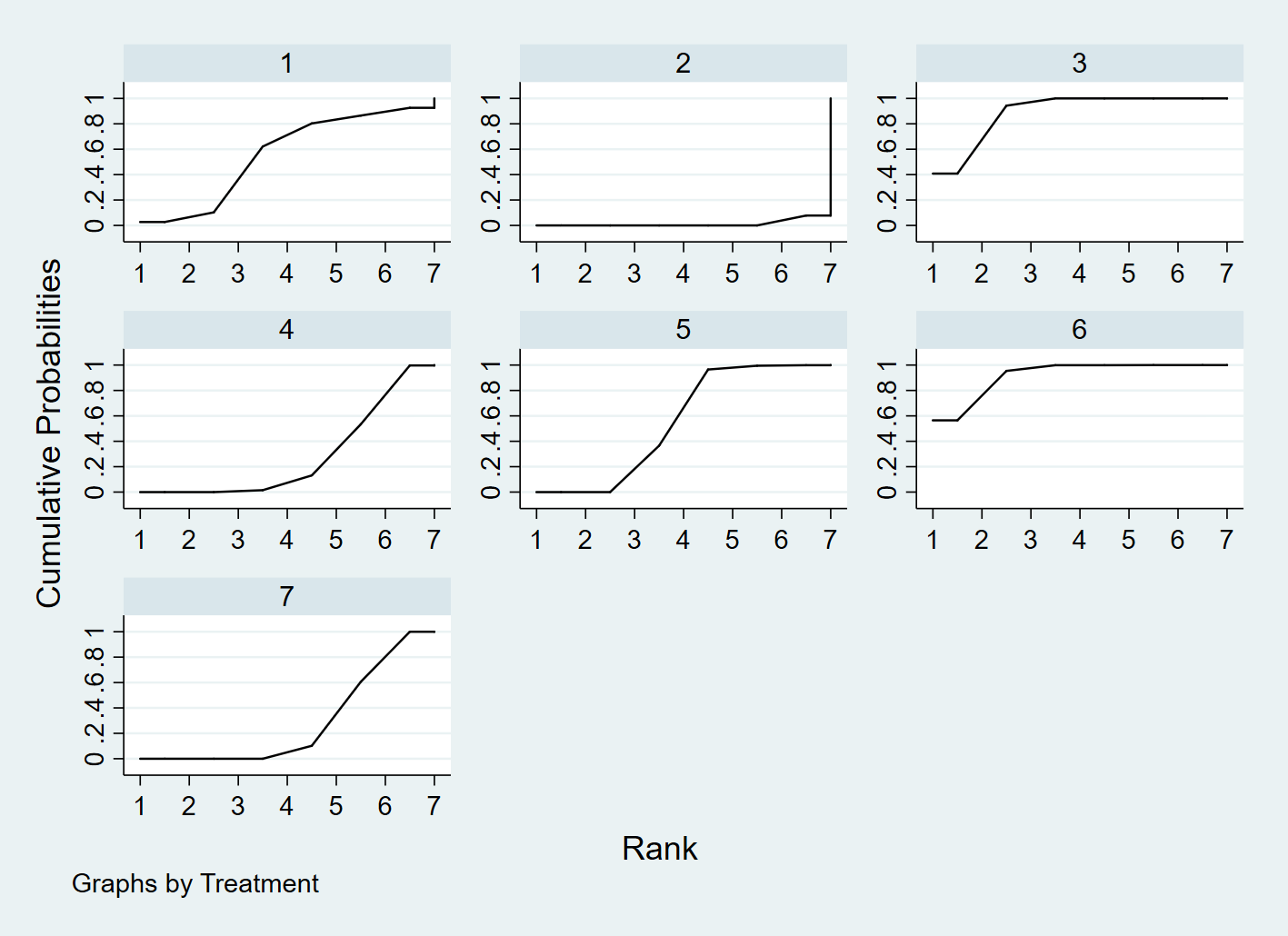
**

Figure4 SUCRA plots of TG in MetS patients with different dietary patterns of intervention(intervention duration: 12 - 48 weeks)

**5. HDL-C**

The results of the network meta-analysis showed that compared with the control diet group, the DASH diet significantly increased the HDL-C level in patients with MetS, as shown in Table 5.

Table 5 League table of all pairwise comparisons of the effects of dietary patterns on HDL-C in MetS patients(intervention duration: 12 - 48 weeks)

| HDL-C | | | | | | |
| --- | --- | --- | --- | --- | --- | --- |
| **DASH diet** |  |  |  |  |  |  |
| **13.74**  **(6.80,20.67)** | **Vegan diet** |  |  |  |  |  |
| **9.71**  **(1.43,17.98)** | -4.03  (-10.09,2.03) | **Low-carbohydrate diet** |  |  |  |  |
| **10.66**  **(3.55,17.78)** | -3.07  (-6.26,0.12) | 0.96  (-5.33,7.24) | **Mediterranean diet** |  |  |  |
| **10.40**  **(2.98,17.82)** | -3.34  (-8.16,1.49) | 0.69  (-2.96,4.35) | -0.26  (-5.35,4.82) | **Low-fat diet** |  |  |
| 8.40  (-1.99,18.78) | -5.34  (-14.06,3.38) | -1.31  (-9.44,6.82) | -2.27  (-11.13,6.60) | -2.00 (-9.26,5.26) | **Ketogenic diet** |  |
| **9.83**  **(3.52,16.13)** | -3.91  (-6.82,-0.99) | 0.12  (-5.27,5.52) | -0.84  (-4.12,2.45) | -0.57 (-4.52,3.37) | 1.43 (-6.84,9.69) | **Control**  **diet** |

The SUCRA ranking results for studies with intervention durations of 12–48 weeks were as follows: vegan diet (SUCRA = 93.7%) > low-fat diet (SUCRA = 59.2%) > Mediterranean diet (SUCRA = 58.1%) > low-carbohydrate diet (SUCRA = 50.3%) > control diet (SUCRA = 46.2%) > ketogenic diet (SUCRA = 41.4%) >DASH diet (SUCRA = 1.1%). The ranking outcomes of each dietary pattern are presented in Figure 5.


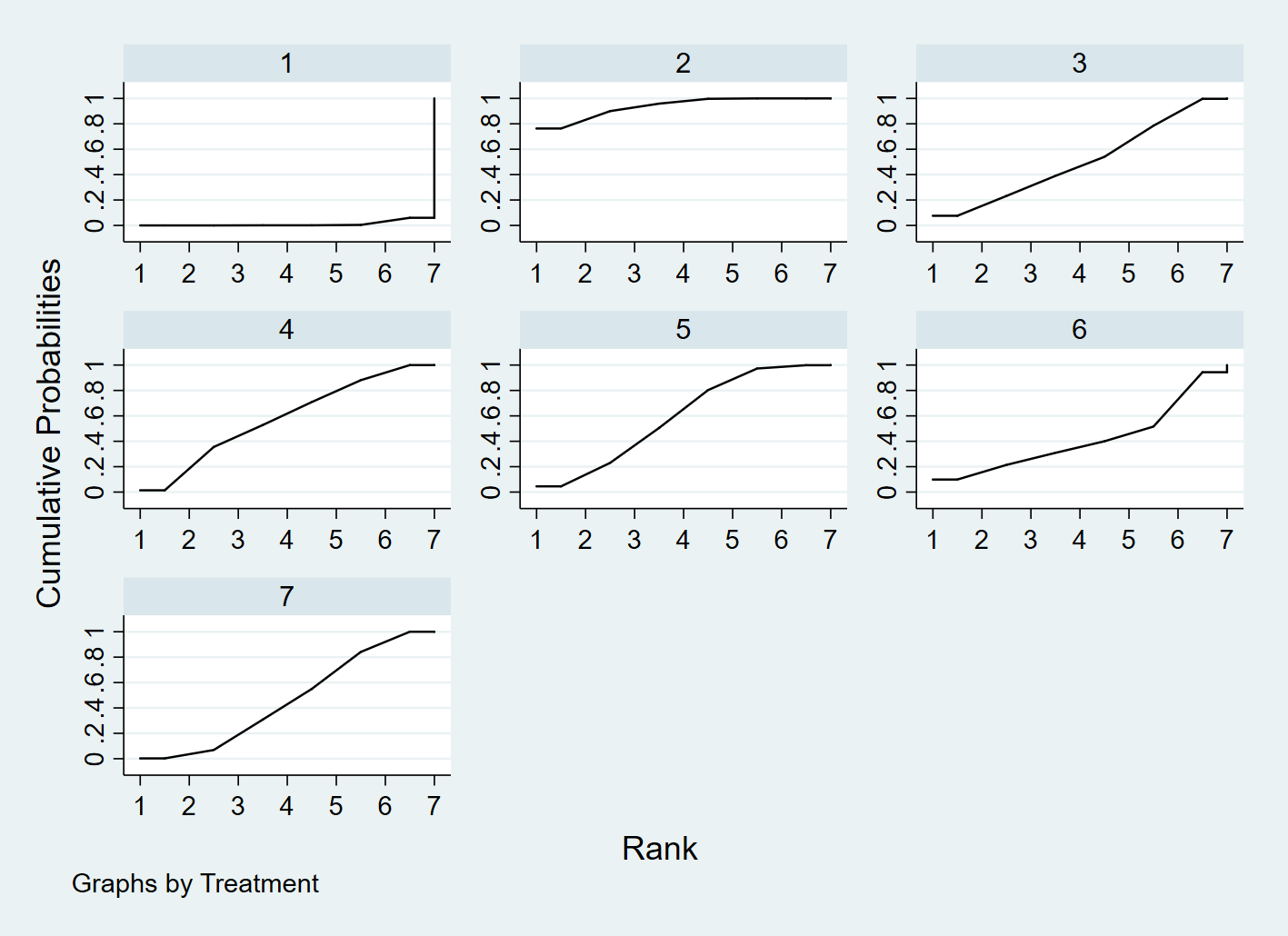


Figure 5 SUCRA plots of HDL-C in MetS patients treated with different dietary patterns(intervention duration: 12 - 48 weeks)

**6.FBG**

The results of the network meta-analysis showed that compared with the control diet group, the vegetarian diet [MD=-0.26, 95%CI (-0.53, -0.01)], low-carbohydrate diet [MD=-0.43, 95%CI (-0.80, -0.06)] and the Mediterranean diet [MD=-0.24, The 95%CI (-0.51, -0.03) significantly controlled the fasting blood glucose of patients with MetS, as shown in Table 6.

Table 6 League table of all pairwise comparisons of the effects of dietary patterns on FBG in MetS patients (intervention duration: 12 - 48 weeks)

| FBG | | | | | |
| --- | --- | --- | --- | --- | --- |
| **DASH diet** |  |  |  |  |  |
| -0.06  (-0.59,0.46) | **Vegan diet** |  |  |  |  |
| 0.11  (-0.47,0.69) | 0.17  (-0.30,0.64) | **Low-carbohydrate diet** |  |  |  |
| -0.08  (-0.60,0.45) | -0.02  (-0.24,0.21) | -0.19  (-0.65,0.28) | **Mediterranean diet** |  |  |
| -0.06  (-0.59,0.46) | -0.00  (-0.40,0.39) | -0.17  (-0.43,0.08) | 0.01  (-0.37,0.40) | **Low-fat diet** |  |
| -0.32  (-0.77,0.13) | **-0.26**  **(-0.53,-0.01)** | **-0.43**  **(-0.80,-0.06)** | **-0.24**  **(-0.51,-0.03)** | -0.25 (-0.52,0.01) | **Control diet** |

The SUCRA ranking results for studies with intervention durations of 12–48 weeks were as follows:low-carbohydrate diet (SUCRA = 81.8%) >DASH diet (SUCRA = 61.4%) > vegan diet (SUCRA = 53.8%)> low-fat diet (SUCRA = 49.6%)>Mediterranean diet (SUCRA = 49.3%) > control diet (SUCRA = 4.1%). The ranking outcomes of each dietary pattern are presented in Figure 6.


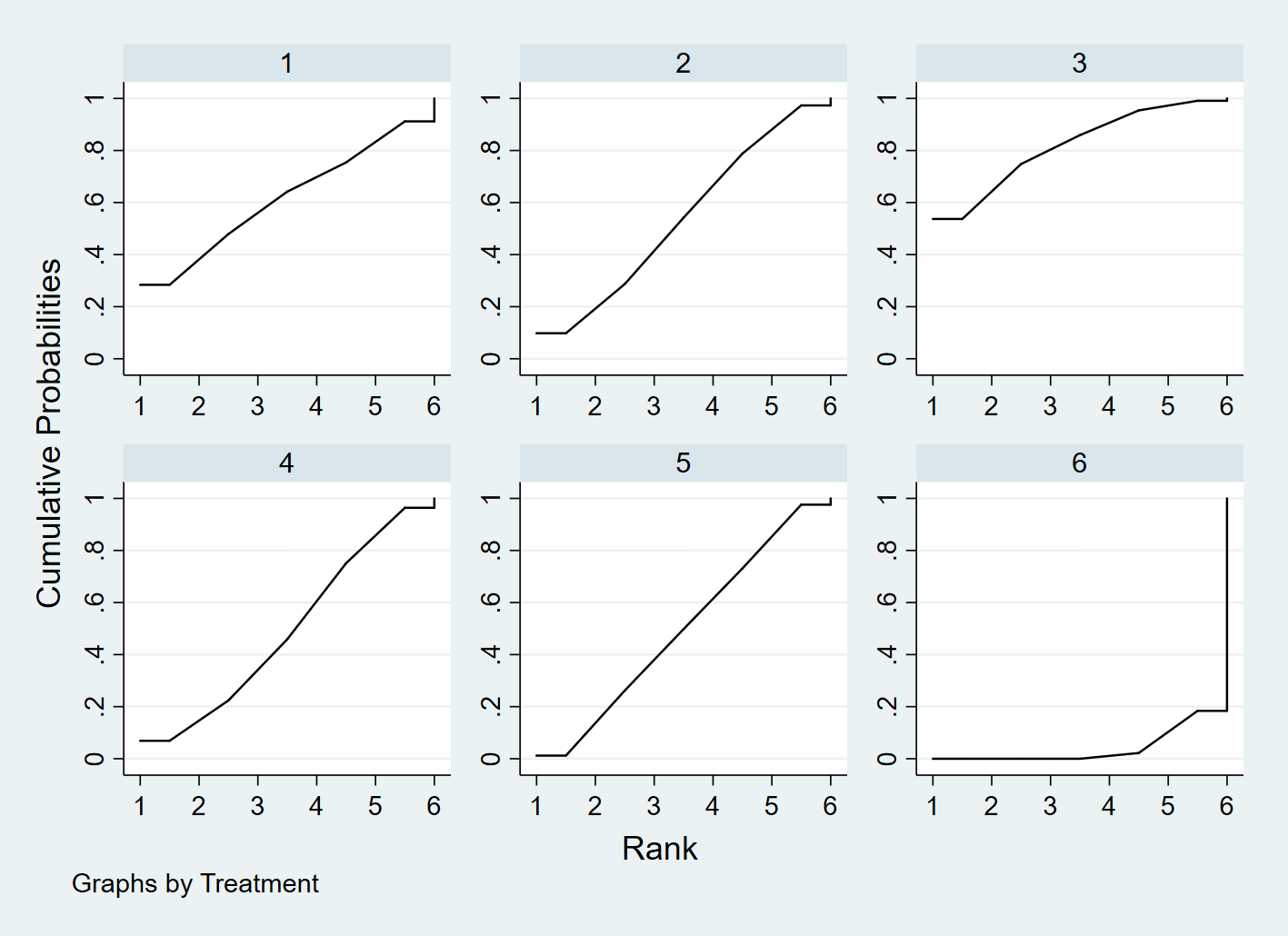


Figure 6 SUCRA plots of FBG in MetS patients treated with different dietary patterns(intervention duration: 12 - 48 weeks)
